# Characterization of *Shigella flexneri* serotype 6 strains from geographically diverse low- and middle-income countries

**DOI:** 10.1128/mbio.02210-24

**Published:** 2024-12-10

**Authors:** Caitlin E. Gabor, Charlotte E. Chong, Jose M. Lemme-Dumit, Tracy H. Hazen, Kate S. Baker, Karen L. Kotloff, Irene N. Kasumba, Sharon M. Tennant, Henry Badji, M. Jahangir Hossain, Richard Omore, Benjamin Ochieng, Alex O. Awuor, Billy Ogwel, Jane Juma, Eileen M. Barry, David A. Rasko

**Affiliations:** 1 Institute for Genome Sciences, University of Maryland School of Medicine, Baltimore, Maryland, USA; 2 Department of Microbiology and Immunology, University of Maryland School of Medicine, Baltimore, Maryland, USA; 3 Center for Vaccine Development and Global Health, University of Maryland School of Medicine, Baltimore, Maryland, USA; 4 Department of Genetics, University of Cambridge, Cambridge, United Kingdom; 5 Department of Pediatrics, University of Maryland School of Medicine, Baltimore, Maryland, USA; 6 Department of Medicine, University of Maryland School of Medicine, Baltimore, Maryland, USA; 7 Medical Research Council Unit, The Gambia at the London School of Hygiene & Tropical Medicine, Banjul, The Gambia; 8 Kenya Medical Research Institute, Center for Global Health Research (KEMRI-CGHR), Kisumu, Kenya; 9 Center for Pathogen Research, University of Maryland School of Medicine, Baltimore, Maryland, USA; Institut Pasteur, Paris, France

**Keywords:** *Shigella*, *Shigella flexneri*, *Shigella flexneri *serotype 6, comparative genomics, host-pathogen interactions

## Abstract

*Shigella flexneri* serotype 6 (*Sf*6) is one of the most common serotypes recovered from surveillance studies of moderate to severe diarrhea. Despite the clinical significance of *Sf*6, this serotype is understudied. In this work, we have performed both serotype-specific genomic and phenotypic comparisons of *Sf*6 isolates to one another and non-*S*. *flexneri* serotypes. Comparative genomic analyses identified significant nucleotide homology between *Sf*6 strains (*n* = 325), despite a broad range of collection timeframes and geographic locations. We identified *Sf*6 specific factors, including a potential novel *Shigella* virulence factor (type II secretion system). Additionally, we identified established *Shigella* virulence genes (*ospG*) and metabolic genes (*rutABCDEFGR*) that were absent in *Sf*6 strains while present in the majority of 728 non-*Sf*6 strains. Complete sequencing of 11 clinical *Sf*6 strains, demonstrated that the *Sf*6 virulence plasmid (pINV) is ~38 kb smaller than the average non-*Sf*6 pINV (~228 kb). Comparisons of *S. flexneri* species level antibiotic susceptibility highlighted that clinical *Sf*6 isolates from Africa in the Global Enteric Multicenter Study (GEMS) and Vaccine Impact on Diarrhea in Africa (VIDA) study demonstrated geographic, serotype-specific susceptibility pattern. Phenotypic analyses of *Sf*6 identified reduced intracellular invasion and cytokine induction from HT-29 cells, as well as reduced Ipa protein effector secretion, compared with *S. flexneri* serotype 2a strain 2457T. Together these data highlight conserved and unique serotype-specific genotypic and phenotypic features for *Sf*6. This level of conservation has not been noted for other *S. flexneri* serotypes and is promising for vaccine and diagnostic assays to provide global *Sf*6-specific coverage.

**IMPORTANCE:**

Shigellosis is an ongoing global public health crisis with >270 million annual episodes among all age groups; however, the greatest disease burden is among children in low- and middle-income countries (LMIC). The lack of a licensed *Shigella* vaccine and the observed rise in antimicrobial-resistant *Shigella* spp. highlights the urgency for effective preventative and interventional strategies. The inclusion of *S. flexneri* serotype 6 (*Sf*6) is a necessary component of a multivalent vaccine strategies based on its clinical and epidemiological importance. Given the genomic diversity of *Sf*6 compared with other *S. flexneri* serotypes and *Sf*6 unique O-antigen core structure, serotype-specific characterization of *Sf*6 is a critical step to inform *Shigella*-directed vaccine and alternative therapeutic designs. Herein, we identified conserved genomic content among a large collection of temporally and geographically diverse *Sf*6 clinical isolates and characterized genotypic and phenotypic properties that separate *Sf*6 from non-*Sf*6 *S. flexneri* serotypes.

## INTRODUCTION

Diarrhea-causing enteric pathogens are responsible for more than half a million childhood deaths annually and >910 million childhood cases of diarrhea per year within low- and middle- income countries (LMICs) (1). *Shigella* spp. are the most prevalent, diarrhea-causing bacterial pathogens recovered from children aged 0–59 months (2, 3). *Shigella* spp. are highly contagious and despite significant public health efforts to improve water quality and sanitation, they are a significant cause for concern (4). One factor that makes the development of a *Shigella* preventative treatment challenging, is the extensive pathogen diversity (5, 6).

*Shigella* spp. are composed of four sub-species. The *Shigella* species predominantly isolated from stool in LMICs are *S. flexneri* and *S. sonnei* (7). *S. flexneri* accounted for 65.9% of *Shigella* spp. isolates within the Global Enteric Multicenter Study (GEMS) (3, 8) and 67.6% in the Vaccine Impact on Diarrhea in Africa (VIDA) study (9). *S. flexneri,* unlike the highly clonal *S. sonnei,* is genetically diverse and is composed of >15 serotypes (7). *S. flexneri* serotypes 1–5, X and Y can be differentiated into seven phylogenomic (PG) groups, which can be further differentiated into genomic clades and sub-clades independent of their serotype designation (5). *S. flexneri* serotype 6 (*Sf*6) separates into a distinct genomic lineage from PG 1–7 (5). *Sf*6 has been found to cause significant disease burden across multiple surveillance studies in LMICs, including GEMS and VIDA (3, 6, 8–10). Therefore, the inclusion of *Sf*6 in multivalent *Shigella* vaccine strategies is necessary.

Despite its clinical significance, there are limited analyses of the unique genomic content and serotype-mediated phenotypes of *Sf*6. Limited historical studies have demonstrated that *Sf*6 is phenotypically different from *S. flexneri* serotypes 1–5: including greater fermentative activity, unique nutritional requirements, and a unique core O-antigen structure not found in other *Shigella* spp. (11–15). To date, large scale *Sf*6 genomic analyses that include patient metadata have been limited to a single country (Bangladesh [16]) or a small region within Asia (17). None of these studies have analyzed geographically and temporally diverse *Sf*6 strains.

We previously reported genomic, transcriptomic, and phenotypic differences among three archetype *S. flexneri* strains (18). The *Sf*6 archetype strain, CCH060, was both genotypically and phenotypically different from the other archetype strains examined. In the current study, we genomically and phenotypically analyze a diverse collection of *Sf*6 strains to each other and to non-*Sf*6 *S. flexneri* serotype strains. Herein, we examined a collection of *Sf*6 strains (*n* = 325) from Africa, Asia, and Europe predominately collected during the GEMS and VIDA studies (3, 8, 9, 17, 19, 20). Overall, these data highlight highly conserved serotype-specific genomic features and phenotypes among *Sf*6 that can be used to inform current therapeutic designs and diagnostic approaches.

## RESULTS

### Description of Sf6 genomes examined in this study

A total of 332 *Sf*6 whole genome sequences (3, 8, 9, 17, 19, 20) were aggregated to examine the genomic content of *Sf*6 strains from diverse geographic regions; seven genomes were omitted as initial analyses determined that they were not phylogenomically similar to the other *Sf*6 strains (Data set 1). The remaining 325 strains include representation from 27 countries on four continents and cover a timeframe of 65 years ranging from 1953 to 2018 (Data set 1).

In addition, we completed the genomes of 11 geographically diverse isolates from GEMS (Table 1) from Africa and Asia, which have a significant burden of *S. flexneri* (including *Sf*6) infections (3, 8). Based on epidemiological burden from each continent in GEMS, isolates from two countries from each continent that had the greatest disease burden were selected (3, 8): Pakistan, Bangladesh, Kenya, and The Gambia. Each of the 11 completed *Sf*6 genomes contained a single chromosome (4.676 ± 0.034 Mb), which is similar in size to our previously sequenced *Sf*6 archetype strain CCH060 (4.96 Mb) (18). The size of the chromosome is similar to publicly available, completed non-*Sf*6 genomes (4.66 ± 0.007 Mb; Table S1).

**TABLE 1:** Characteristics of complete *S.flexneri* serotype six genome assemblies

Isolate characteristics			Assembly characteristics										
Isolate	Geographic location	*Sf*6 clade	No. of contigs	Genome size (bp)	Overall GC%	Contig label	Contig description	Sequence length (bp)	GC%	Plasmid incompatibility types	AMR genes (acquired)	GenBank accession no.	SRA accession no.
703427	Pakistan (Asia)	*Sf*6_V	6	4,896,707	51.00	703427	Chromosome	4,699,891	51.23	None	*2x aadA, sat2, dfrA1, tetB, catA1, bla_OXA-1_*	CP162080	SRR29762673, SRR29762670
						p703427_190	Virulence plasmid	190,780	45.20	IncFII(AY458016)	None	CP162081	
						p703427_6	Cryptic plasmid	6,036	53.78	None	*sul2*	CP162082	
						p703427_4	Cryptic plasmid	4,065	53.16	None	None	CP162083	
						p703427_2a	Cryptic plasmid	2,783	42.01	None	None	CP162084	
						p703427_2b	Cryptic plasmid	2,679	45.73	None	None	CP162085	
700302	Pakistan (Asia)	*Sf*6_V	7	5,010,188	50.91	700302	Chromosome	4,718,931	51.20	None	*2x aadA, sat2, dfrA1, tetB, catA1, bla_OXA-1_*	CP162073	SRR29762672, SRR29762669
						p700302_193	Virulence plasmid	193,303	45.31	IncFII(AY458016)	None	CP162074	
						p700302_90	Cryptic plasmid	90,188	47.68	None	None	CP162075	
						p700302_7	Cryptic plasmid	7,766	55.12	None	*sul2*	CP162076	
						p700302_2a	Cryptic plasmid	2,783	42.01	None	None	CP162077	
						p700302_2b	Cryptic plasmid	2,679	45.73	None	None	CP162078	
						p700302_1	Cryptic plasmid	1,780	57.02	ColpVC(JX133088)	None	CP162079	
702865	Pakistan (Asia)	*Sf*6_V	7	4,935,446	50.97	702865	Chromosome	4,723,129	51.20	None	*2x aadA, sat2, dfrA1, tetB, catA1, bla_OXA-1_*	CP162066	SRR29762661, SRR29762668
						p702865_190	Virulence plasmid	190,801	45.21	IncFII(AY458016)	None	CP162067	
						p702865_15	Cryptic plasmid	15,480	53.52	None	None	CP162068	
						p702865_6	Cryptic plasmid	6,036	53.76	None	*sul2*	CP162069	
						p702865_4	Cryptic plasmid	4,065	53.16	None	None	CP162070	
						p702865_2a	Cryptic plasmid	2,783	41.97	None	None	CP162071	
						p702865_2b	Cryptic plasmid	2,679	45.73	None	None	CP162072	
604193	Bangladesh (Asia)	*Sf*6_VI	7	4,890,254	50.99	604193	Chromosome	4,695,879	51.22	None	*tetB*	CP162059	SRR29762658, SRR29762667
						p604193_187	Virulence plasmid	187,596	45.22	IncFII(AY458016)	None	CP162060	
						p604193_7	Cryptic plasmid	6,779	52.01	None	*sul2, dfrA14, aph (6)-Id*	CP162061	
						p604193_4	Cryptic plasmid	4,065	53.16	None	None	CP162062	
						p604193_2	Cryptic plasmid	2,679	45.73	None	None	CP162063	
						p604193_1a	Cryptic plasmid	1,976	56.28	ColpVC(JX133088)	None	CP162064	
						p604193_1b	Cryptic plasmid	1,546	51.49	Col(MG828)	None	CP162065	
600006	Bangladesh (Asia)	*Sf*6_VI	6	4,868,275	50.99	600006	Chromosome	4,666,868	51.22	None	*tetB*	CP162053	SRR29762657, SRR29762666
						p60006_188	Virulence plasmid	188,114	45.25	IncFII(AY458016)	None	CP162054	
						p60006_13	Cryptic plasmid	13,293	49.53	None	None	CP162055	
						p60006_4	Cryptic plasmid	4,065	53.16	None	None	CP162056	
						p60006_2	Cryptic plasmid	2,679	45.73	None	None	CP162057	
						p60006_1a	Cryptic plasmid	1,546	51.49	Col(MG828)	None	CP162058	
600717	Bangladesh (Asia)	*Sf*6_V	5	4,838,555	51.00	600717_1	Chromosome	4,651,652	51.24	None	*aadA, sat2, dfrA1, gyrA(D87Y*)	CP162048	SRR29762665, SRR29762656
						p600717_186	Virulence plasmid	186,903	45.18	IncFII(AY458016)	None	CP162049	
						p600717_4	Cryptic plasmid	4,065	53.16	None	None	CP162050	
						p600717_2 a	Cryptic plasmid	2,783	42.01	None	None	CP162051	
						p600717_2b	Cryptic plasmid	2,679	45.69	None	None	CP162052	
603020	Bangladesh (Asia)	*Sf*6_VI	6	4,874,316	50.98	603020	Chromosome	4,683,211	51.21	None	*tetB*	CP162042	SRR29762655, SRR29762664
						p603020_184	Virulence plasmid	184,337	45.08	IncFII(AY458016)	None	CP162043	
						p603020_7	Cryptic plasmid	6,768	52.05	None	*sul2, dfrA14, aph (6)-Id*	CP162044	
						p603020_4	Cryptic plasmid	4,066	53.15	None	None	CP162045	
						p603020_2	Cryptic plasmid	2,679	45.73	None	None	CP162046	
						p603020_1 a	Cryptic plasmid	1,546	51.49	Col(MG828)	None	CP162047	
102751	The Gambia (Africa)	*Sf*6_VII	5	4,930,867	50.99	102751	Chromosome	4,637,453	51.25	None	None	CP160651	SRR29762654, SRR29762663
						p102751_60	Virulence plasmid_1	60,981	47.50	IncFII(AY458016)	None	CP160652	
						p102751_118	Virulence plasmid_2	118,944	43.93	None	None	CP160653	
						p102751_12	Virulence plasmid_3	12,456	47.08	None	None	CP160654	
						p102751_100	Plasmid	100,996	50.38	IncB/O/K/Z(GU256641)	*aadA, sat2, dfrA1, tetB, sul2*	CP160655	
100537	The Gambia (Africa)	*Sf*6_VII	2	4,879,772	51.01	100537	Chromosome	4,694,836	51.23	None	*tetB*	CP160649	SRR29762653, SRR29762662
						p100537_184	Virulence plasmid	184,936	45.42	IncFII(AY458016)	None	CP160650	
401192	Kenya (Africa)	*Sf*6_IV	2	4,831,903	51.03	401192	Chromosome	4,635,312	51.26	None	None	CP160647	SRR29762652, SRR29762652
						p401192_196	Virulence plasmid	196,591	45.46	IncFII(AY458016)	None	CP160648	
403127	Kenya (Africa)	*Sf*6_IV	2	4,826,393	51.02	403127	Chromosome	4,631,379	51.26	None	None	CP160645	SRR29762671, SRR29762659
						p403127_195	Virulence plasmid	195,014	45.43	IncFII(AY458016)	None	CP160646	

All 11 completed *Sf*6 strains contained a pINV plasmid (21), encoding the ~30 kb *mxi-spa-ipa* pathogenicity island, as a single contig with the exception of *Sf*6 strain 102751 in which the locus was encoded on three different contigs (Table 1). The size of the completed pINV for the *Sf*6 genomes are 190 ± 4.2 kb, which is significantly smaller (Mann–Whitney U test; *P* < 0.00001) than the average non-*Sf*6 pINV (227 ± 9.9 kb; Table S1), in agreement with previous reports (22, 23).

### Phylogenomic characterization of 325 *Sf*6 genomes from diverse geographic origins

**Fig 1:**
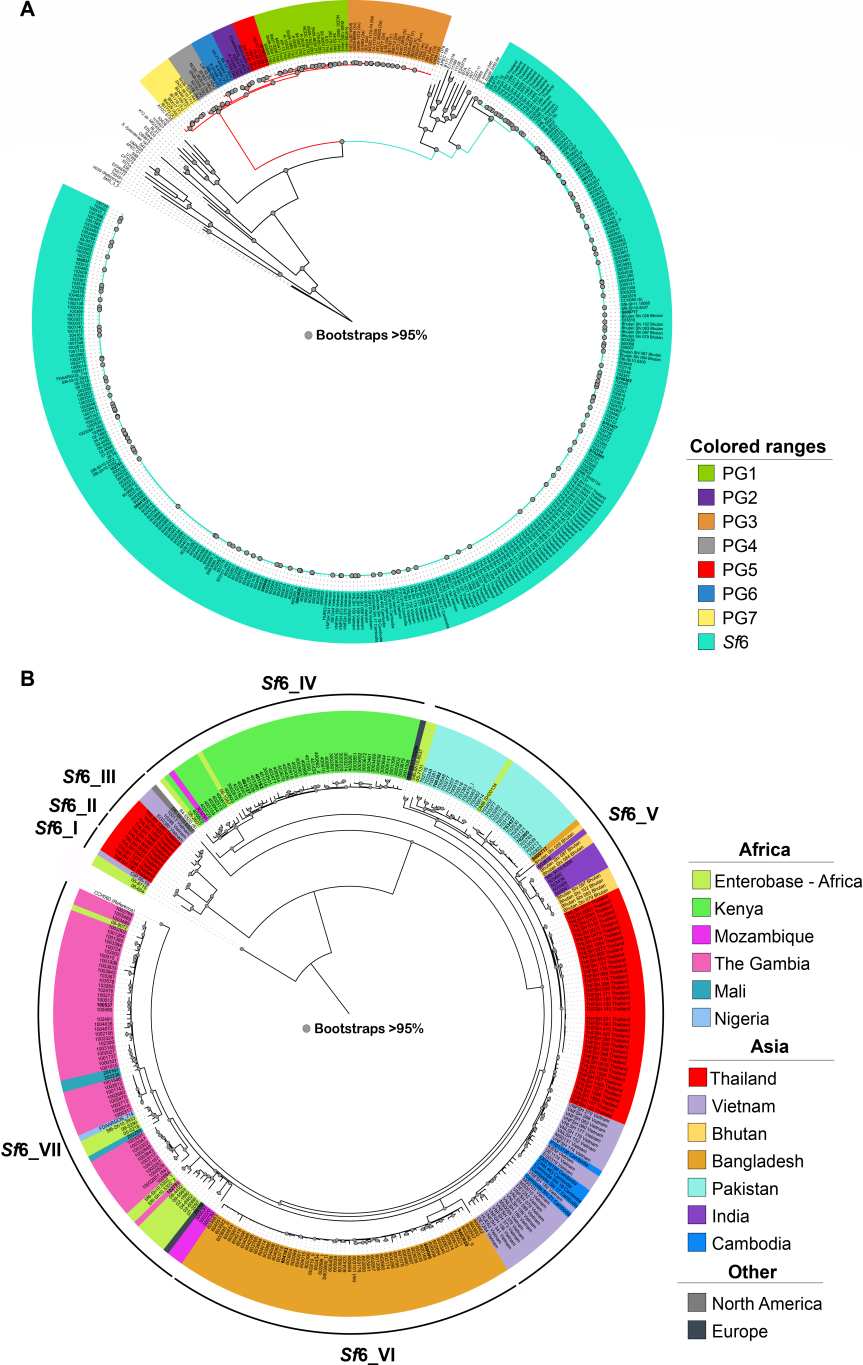
Genomic relationships of 325 *Sf*6 geographically diverse genomes. (A) Diverse *E. coli* and *Shigella* genomes (26) were compared with 325 *Sf*6 genomes (teal) (Data set 1). A total of 158,917 SNPs were determined relative to the *E. coli* IA139 (NC_011750.1) reference. The phylogenomic groups are designated PG1 to PG7 (see legend), as described previously (5), and the serotypes, where known, are indicated in parentheses next to each strain name. *S. flexneri* lineage delineated by color; Lineage 1 (red) containing PG 1–7 and Lineage 2 (teal) containing *Sf*6. (B) All *Sf6* strains (*n* = 325) were compared with *Sf6* archetype strain CCH060 (CP099865, CP099866, CP09867). A total of 6,904 SNPs were determined relative to *Sf*6 reference. Colors were used to indicate country of origin for each isolate (see legend). The *Sf*6 clades are designated *Sf*6_I-*Sf*6_VII as indicated by major branch points of genomic similarity (by SNP and BAPS comparisons) as indicated by the outmost, black ring are based on the number of SNPs identified in the phylogenomic analysis (as described in the Material and Methods). The *Sf*6 genomes completed in this study are indicated in bold. The genomes included are listed in Supplemental Data set 1.

A whole-genome phylogeny was inferred from the *Sf*6 genomes along with representative *S. flexneri*, *Escherichia coli*, and other *Shigella* spp. genomes (Dataset 1). All the *Sf*6 strains formed a single distinct lineage (Fig. 1A), congruent with previous analyses (24). Assessment of the genomic diversity between only the *Sf*6 genomes using both SNP number and BAPS (25) revealed that *Sf*6 strains formed seven clades: *Sf*6_I – *Sf*6_VII (Fig. 1B). The majority of *Sf*6 genomes in this study were within *Sf*6_IV - *Sf*6_VII. The average number of SNPs among strains within these clades is less than 100 (74 ± 35.2 SNPs). The *Sf*6 strains within clades *Sf*6_I – *Sf*6_III had the greatest genetic diversity (269 ± 232.5 SNPs). Additionally, clades *Sf*6_I - *Sf*6_III had greater diversity between themselves and clades *Sf*6_IV -*Sf*6_VII (941 ± 31.2 SNPs). However, the overall SNP distances between the *Sf*6-designated clades were relatively small (max <1,000 SNPs), highlighting remarkable genomic conservation among *Sf*6.

Within the phylogeny, we observed a geographic distribution (Fig. 1B). For example, clade *Sf*6_IV contained all strains from Kenya, and *Sf*6_VI contained all strains from Bangladesh. The remainder of the strains isolated from Africa were within clade *Sf*6_VII, and those from Asia were in clade *Sf*6_V and *Sf*6_I - *Sf*6_III. Geographic groupings included temporally diverse samples, as both GEMS and VIDA isolates from Africa grouped together by country of origin even though there is upwards of 10 years between isolates (2008–2018).

### Genomic differences among *Sf*6 and other *S. flexneri* serotypes

As previously noted, *Sf*6 is genomically distinct from the other *S. flexneri* serotypes (5, 11). However, a detailed large-scale analysis of the unique *Sf*6 genomic content has not been performed. To identify *Sf*6 serotype-specific genomic features, we compared the predicted protein-coding gene content of *Sf*6 vs non-*Sf*6 genomes representing all other *S. flexneri* serotypes (Data set 2). Of the 20,812 genes predicted across all *S. flexneri* genomes, only 4.3% (899/20,812) were shared by 100% of the *S. flexneri* strains. These shared genes included several central metabolic factors, including the phosphotransferase (PTS) system and nitrate metabolism, as well as ribosomal and replication related genes.

Among the *Sf*6 unique genomic content, 68 genes were identified in all *Sf*6 strains and absent from all non-*Sf*6 strains (Data set 2). Genes identified included intrinsic antimicrobial resistance genes (ARGs) (*emrA*, *mdtM*, and *mdtE*; Data set 2) and type II secretion system (T2SS) components (Table 2). Of the 325 *Sf*6 genomes, 100% contained 13 putative genes for a T2SS (*gspC2_1, gspD2_2, gspD2_1, epsE_2, epsE_3, epsF_2, epsG, epsH, epsI, epsJ, gspK, epsL,* and *epsM*), and 66.2% (215/325) contained one putative gene (*gspC2_2*) located next to *gspC2_1* in our completed genomes. There are three genes (*gspC, gspD* and *gspE*) within the T2SS gene cluster that appear to be pseudogenes. The genes encoding the T2SS while not present within the other *S. flexneri* serotypes have been previously identified in *S. dysenteriae* serotype 1 strain 197 and *S. boydii* serotype 4 strain Sb227 (27); however, the T2SS was not functionally characterized.

**TABLE 2:** Comparative genomics of *Sf6* and non-*Sf6 S. flexneri* serotypes^*a*^^,^^*b*^^,^^*c*^

Gene	Function	*Sf6* (*n* = 325)		Non-*Sf6* (*n* = 728)	
		Percent present in:	Genomic comparison results:	Percent present in:	Genomic comparison results:
Virulence genes					
T2SS genes	Type II secretion system (T2SS); secretion of virulent and non-virulent effectors	**92.0% (299**)^*f*^	*gspC;* predicted frameshift^*d*^ *gpsD;* predicted frameshift^*d*^	0.0% (0)	Absent
*ospG*	Downregulation of host innate immune response	0.0% (0/310)^*e*^	Absent	96.3% (558/579)^*e*^	Intact
Metabolism genes					
*fucP*	L-fucose/proton symporter	**group_5343; 96.3% (313**)*fucP;* 0.0% (0)	group_5343; missing first 342 nt	group_5343; 3.2% (23). ***fucP*; 96.3% (701**)	*fucP*; Intact
*fucI*	L-fucose isomerase	**group_4920; 90.7% (295**) *fucl*; 3.4% (11)	group_4920; Missing aa 352–355	group_4920; 5.9% (43) ***fucl*; 96.7% (704**)	*fucl*; Intact
*fucO*	L-1,2-propanediol oxidoreductase	**group_6231; 89.8% (292**) *fucO*; 5.8% (19)	group_6231; 10 SNPs and shorter by 1 aa	group_6231; 19.3% (141) ***fucO*; 81.0% (590**)	*fucO*; Intact
*rutABCDEFGR*	Pyrimidine utilization	0.0% (0)	Absent; contains *rutR* regulator only	**86.2%. (689**)	Intact
Fimbriae genes					
*fimA*	Type 1 fimbriae protein, A chain	**group_14286; 99.7% (324**)*fimA*; 0.0% (0)	97% homology to *E. coli* K12 aa (NC_000913.3)	group_14286; 0.0% (0) ***fimA*; 99.0% (721**)	91% homology to *E. coli* K12 aa (NC_000913.3)
Other					
*pemI*	Antitoxin; part 1 of *pemI*, *pemk* toxin/antitoxin system	**100% (325**)	73% homology to Ec P13975.1	0.0% (0)	Absent
*pemk*	Toxin; part 2 of *pemI*, *pemk* toxin/antitoxin system	*pemk*; 0.0%. (0) **group_12476; 100% (325**)	71% homology to Ec P13975.1	***pemk*; 98.6% (718**) group_12476; 0.0% (0)	35% homology to Ec P13975.1
*mazEF*	Toxin/antitoxin; suicide module in response to stress	**100% (325**)	Intact	0.0% (0)	Absent
*ompC*	Outer membrane protein C	**group_14161; 100% (325**)*ompC*; 0.0% (0)	3 aa longer and 26 nt variations	group_14161; 0.0% (0) ***ompC*; 99.8% (727**)	Intact; 100% identity to 301T (GCA_000006925.2)

^*a*^ Intact: 100% alignment of nt to reference gene.

^*b*^ Absent: BLAST and Roary no gene present in genome.

^*c*^ nt: nucleotide, aa: amino acid.

^*d*^ Predicted frameshift from GenBank CCH060 annotation.

^*e*^ Only genomes with required pINV genes were compared (must contain *mxi-spa-ipa operon, repA, repB, virB*, and *virF*).

^*f*^ The bold formatting indicates differences in prevalence among the genes identified in the table.

Among non-*Sf*6 genomes, there were 26 genes present in 100% of non-*Sf*6 genomes and absent in all *Sf*6 genomes (Data set 2). These included three metabolic genes for L-fucose utilization and the nine genes that are contained within the pyrimidine utilization operon (*rutABCDEFGR*) (Table 2). Previously, we noted that *ospG*, a pINV virulence factor that represses the host innate immune response upon invasion (28), was absent from *Sf6* strain CCH060 (18). In our current analysis, *ospG* was absent in all *Sf*6 genomes, whereas it was present in 96% (558/579) of non-*Sf*6 genomes (Data set 3; Table 2).

### Clade and geographic-associated genomic content of *Sf*6

We further examined the genomic diversity among *Sf*6 isolates alone by geographic region (continent or country) and/or each phylogenomic clade. The majority of *Sf*6 genomes analyzed were from two continents: Africa (*n* = 127) and Asia (*n* = 195). There were only two predicted protein-coding genes, encoding a hypothetical protein and inner membrane transporter, that were unique to the *Sf*6 from Asia and no unique genes identified from the *Sf*6 from Africa (Table 3). This was not surprising as *Sf*6 from each continent was distributed throughout the phylogeny (Fig. 1B). However, when grouped by country of origin, we identified several country-associated genes (Table 3).

**TABLE 3:** Comparative genomics of *S. flexneri* serotype 6 isolates

	Number of isolates	Genes present^*a*^	Genes absent^*a*^
Continent			
Africa	127	0	0
Asia	195	2	0
Country			
Bangladesh	63	8	21
Bhutan	7	30	9
Cambodia	6	9	5
India	6	28	12
Kenya	46	11	29
Mali	3	25	23
Mozambique	4	9	6
Pakistan	29	2	9
Thailand	53	3	1
Thailand - *Sf*6_V	42	12	3
Thailand - *Sf*6_I-III	11	68	74
The Gambia	54	7	7
Vietnam	32	8	6
Clade			
*Sf*6_I-III	15	64	72
*Sf*6_IV	49	12	56
*Sf*6_V	63	0	0
*Sf*6_VI	61	7	21
*Sf*6_VII	73	3	6

^*a*^ Genes present were calculated by identifying genes present in ≥80% of isolates from group 1 (i.e., continent, country, or clade) and absent from ≥80% of group 2 (i.e., everything else). The reverse was used to determine genes absent.

Of the *Sf*6 genomes analyzed from countries in Africa, Kenya (*n* = 46), and The Gambia (*n* = 54) had the greatest sample sizes. Within the strains from Kenya, there were 40 distinct genes (Table 3). Three genes that were uniquely absent within *Sf*6 from Kenya have predicted contributions to mannitol utilization (29): *mtlA_2*, *mtlD*, and *mtlR* (Data set 2). However, these strains possess an alternative allelic version of *mtlA* (*mtlA_1*) and *mtlD* (*yggP_1*), which was present in all *Sf*6 strains. It was unclear whether the absence of *mtlA_*2, *mtl*D, and *mtl*R among the strains from Kenya affects overall mannitol utilization. Phenotypically, *S. flexneri* is considered positive for mannitol fermentation (30); however, previous studies have described two biotypes of *Sf*6 (Newcastle and Manchester) that have different mannitol fermentation results (12, 13, 16, 31, 32). The phenotypic differences in carbohydrate utilization among *Sf*6 biotypes may be related to the absence of these genes.

Of the *Sf*6 genomes analyzed from countries in Asia, Bangladesh (*n* = 63) and Thailand (*n* = 53) had the greatest sample sizes. Among the strains from Bangladesh, there were 29 distinct genes (Table 3). In *Sf*6, two allelic versions of *ushA* were predicted, *ushA*_1 and *ushA*_2, which are 76.4% identical at the amino acid level. In *E. coli, ushA* was predicted to be a periplasmic UDP-sugar hydrolase (33), which is secreted and localized to the periplasm where it can degrade UDP-glucose (34). Analysis of our completed *Sf*6 genomes identified *ushA*_1 present on the chromosome, while *ushA*_2 is on pINV. Within all the strains from Bangladesh, the predicted protein-coding gene *ushA_2* is absent (Data set 2). It is unclear how the absence of plasmid-encoded *ushA* will functionally impact the strains from Bangladesh.

Overall, there was little association of *Sf6* gene content with geographic location, which may relate to the wide distribution and lack of geographic restriction of *Sf6*.

### Genomic content of the pINV differs between *Sf*6 and other *S. flexneri* serotypes

Given the genomic distinction of *Sf*6, we sought to determine whether *S. flexneri* differed with respect to major virulence determinants. In *Shigella* spp*.,* several virulence genes are located on the pINV (21). As we noted above, the *Sf*6 pINV (190 ± 4.2 kb) is ~37 kb smaller than the average non-*Sf*6 pINV (227 ± 9.9 kb). To identify the specific genes lacking within the *Sf*6 pINV, we compared previously characterized *Shigella* pINV virulence genes to both *Sf*6 and non-*Sf*6 genomes (Data set 3). Other than *ospG* (Table 2), *sepA*, a virulence factor involved in tight junction loosening and increased invasion (35), was absent in all *Sf*6 (Data set 3) consistent with our previous archetype findings (18). Several virulence genes encoded on the prototypical pINV from *S. flexneri* serotype 5a pWR100 (AL391753), the first sequenced virulence plasmid from *S. flexneri* (36), were also absent from all *Sf*6 genomes: *phon1,* a periplasmic acid phosphatase whose physiological role remains to be determined (37) and plasmid stability proteins *stbB* and *stbA* (Data set 3). These three genes (*phon1*, *stbB*, and *stbA*) are contained within a 8 kb contiguous region on pWR100 (33).

**Fig 2:**
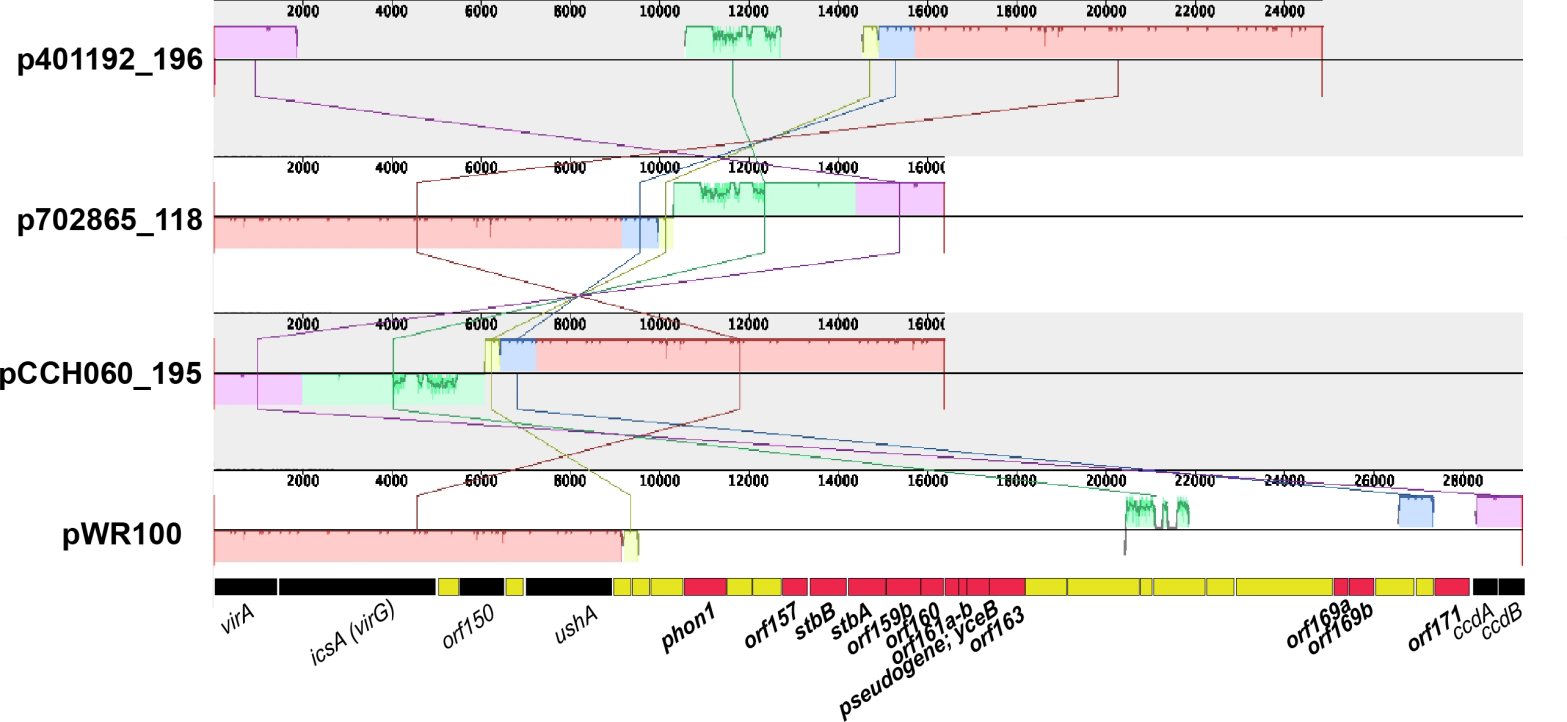
Virulence plasmid comparison of *Sf*6 strains. Nucleotide sequences encompassing *virA- ccdA,B* were aligned from *Sf*5 strain M90T plasmid pWR100 (AL391753; nucleotides 144615–173950) and the plasmids from *Sf*6 strains CCH060 (pCCH060_195 (CP099866); nucleotides 175010–160158), 702865 (p702865_118; nucleotides 154258–170617), and 401192 (p401192_196; nucleotides 0–10288, 196515–183601) using Mauve v.2025.2.5 (38). Genes labeled for pWR100: red = genes missing from *Sf*6 genomes, black = genes shared with *Sf*6 genomes, yellow = insertion elements.

Sequence alignment (38) of the region encompassing *virA* to *ccdA,B* from pINV of three completed *Sf6* strains (Table 1) and *S. flexneri* strain M90T’s pINV (pWR100) revealed the absence of ~18,000 bp from the *Sf*6 plasmids compared with pWR100 (Fig. 2). The first ~9,000 bp of pWR100 including *virA-ushA* (light red boxes; Fig. 2) and *ccdA-ccdB* (purple box; Fig. 2) was conserved among the four *Sf*6 strains; however, the region encompassing genes *phon1-orf171* (red boxes of pWR100 gene map) was absent from the four *Sf*6 strains. BLASTN large-scale BLAST score ratio (LS-BSR) was used to compare the presence or absence of these genes from non-completed *S. flexneri* genomes, as previously described (39, 40). LS-BSR analysis revealed that *orf157* was lacking from 100% of *Sf*6 genomes, *orf159b–orf163* was missing from 95% (307/325), *orf169a-b* was missing from 6% (20/325)*,* and *orf171* was missing from 100%, along with the pINV genes described above (Data set 3). While the absence of some of these pWR100 genes is not exclusive to *Sf*6, they are more commonly present among non-*Sf*6 pINV (90%, 523/579) (Data set 3).

Overall, the *Sf*6 pINV differences compared here underscore our comparative genome findings and stress the conservation of genomic features present or absent among *Sf*6 that distinguish them other *S. flexneri* serotypes.

### Antimicrobial resistance and susceptibility differ between *Sf*6 and other *S. flexneri* serotypes

*Shigella* has acquired increasing numbers of ARGs, which reduces therapeutic options (41). To date, antimicrobial resistance (AMR) for *S. flexneri* has been characterized at the species but not a serotype level (6). Understanding both the geographic and serotype ARG distribution could support prescribing patterns for endemic areas. Within GEMS, *S. flexneri* serotype 2a (*Sf*2a) compromises a disproportionate amount of *S. flexneri* clinical cases (3, 8) (20.2%; 228/745), and this large, single serotype sample size combined with serotype diversity may obfuscate serotype-specific resistance patterns. To explore this, we conducted a retrospective comparison of ARGs among GEMS *S. flexneri* serotypes, comparing *Sf*6, *Sf*2a, and non-*Sf*6 cases.

We discovered that there were 24 acquired ARGs (Table 4) previously identified among *S. flexneri* at the species level (6). Overall, non-*Sf*6 and *Sf*2a GEMS strains contained more resistance genes than *Sf*6 strains. These included genes implicated in resistance to aminoglycosides (*aadA*, *aph(3'')-Ib*), tetracyclines (*tetB*), streptothricins (*sat2*), and β-lactams (*bla*_OXA-1_). Sulfonamide resistance gene, *sul2*, was widespread and found in all *S. flexneri* serotypes. Similarly, trimethoprim resistance genes were widespread in all *S. flexneri* serotypes though the trimethoprim genes present differed by serotype.

**TABLE 4:** *Sf*6 vs. non-*Sf*6 antimicrobial resistance patterns^*a*^

Antimicrobial resistance gene (ARG) distribution					Phenotypic susceptibility distribution						
Gene	Antibiotic class	*S. flexneri* GEMS ARG, Africa and Asia^*c*^			Antibiotic	*S. flexneri* GEMS, Africa^*d*^			*S. flexneri* VIDA, Africa^*d*^		
		Non-*Sf*6 (*n* = 658)	*Sf6 (n = 150)*	*Sf2a (n = 234)*		Non-*Sf*6 (*n* = 134)	*Sf*6 (*n* = 44)	*Sf*2a (*n* = 48)	Non-*Sf*6 (*n* = 240)	*Sf*6 (*n* = 60)	*Sf*2a (*n* = 110)
*aac(3)-IIe*	Aminoglycoside	0.2% (1)	0.0% (0)	0.0% (0)	N/A^*f*^	N/A	N/A	N/A	N/A	N/A	N/A
*aadA*	Aminoglycoside	**79.9% (526)^*e*^**	25.3% (38)	**96.2% (225)**							
*aadA5*	Aminoglycoside	0.0% (0)	0.0% (0)	0.0% (0)							
*aph(3'')-Ib*	Aminoglycoside	**49.7% (327)**	7.3% (11)	**66.2% (155)**							
*aph(6)-Id*	Aminoglycoside	66.0% (434)	66.7% (100)	**84.6% (198)**							
*catA1*	Chloramphenicol	0.0% (0)	0.0% (0)	0.0% (0)	N/A	N/A	N/A	N/A	N/A	N/A	N/A
*tetA*	Tetracycline	8.2% (54)	7.3% (11)	1.7% (4)	N/A	N/A	N/A	N/A	N/A	N/A	N/A
*tetB*	Tetracycline	**83.7% (551)**	70.0% (105)	**96.6% (226)**							
*sat2*	Streptothricin (nucleoside)	**60.2% (396)**	24.7% (37)	**78.2% (183)**	N/A	N/A	N/A	N/A	N/A	N/A	N/A
*mphA*	Macrolide	0.8% (5)	2.0% (3)	0.4% (1)	Azithromycin	**S 100.0% (134)**	**S 100.0% (44)**	**S 100.0% (48)**	**S 94.2% (226)** R 5.8% (14)	**S 80.0% (48)** R 20.0% (12)	**S 93.6% (103)** R 6.3% (7)
*bla_CTX-M-15_*	Beta-lactamase	0.3% (2)	0.7% (1)	0.0% (0)	Ampicillin	S 16.4% (22) **R 83.5% (112)**	**S 100.0% (44)** R 0.0% (0)	S 0.0% (0) **R 100.0% (48)**	S 25.8% (62) I 0.8% (2) **R 73.3% (176)**	**S 83.3% (50)** I 8.3% (5) R 8.3% (5)	S 4.5% (5) **R 95.5% (105)**
*bla_OXA-1_*	Beta-lactamase	**64.3% (423)**	18.7% (28)	**85.0% (199)**							
*bla_OXA-129_*	Beta-lactamase	0.0% (0)	0.7% (1)	0.0% (0)							
*bla_TEM-1_*	Beta-lactamase	7.6% (50)	7.3% (11)	1.3% (3)							
*sul1*	Sulfonamide	1.4% (9)	6.1% (10)	0.4% (1)	Trimethoprim-sulfonamide (SXT)	S 13.4% (18) **R 86.6% (116)**	S 2.3% (1) **R 97.8% (43)**	S 12.5% (6) **R 87.5% (42)**	S 7.1% (17) I 0.4% (1) **R 92.5% (222)**	S 3.3% (2) **R 96.7% (58)**	S 4.5% (5) **R 95.5% (105)**
*sul2*	Sulfonamide	72.6% (478)	85.3% (128)	85.0% (199)							
*dfrA1*	Trimethoprim	**68.1% (448)**	24.6% (37)	**79.0% (185)**							
*dfrA14*	Trimethoprim	21.7% (143)	**61.3% (92)**	18.4% (43)							
*dfrA15*	Trimethoprim	0.8% (5)	0.7% (1)	0.0% (0)							
*dfrA17*	Trimethoprim	0.2% (1)	0.0% (0)	0.0% (0)							
*dfrA21*	Trimethoprim	0.0% (0)	0.7% (1)	0.0% (0)							
*dfrA5*	Trimethoprim	0.8% (5)	0.0% (0)	0.9% (2)							
*dfrA7*	Trimethoprim	0.2% (1)	5.3% (8)	0.0% (0)							
*qnrs1*	Fluroquinolone	3.8% (25)	3.3% (5)	0.9% (2)	Nalidixic acid	S 54.5% (73) R 45.5% (61)	S 34.1% (15) I 2.3% (1) **R 63.6% (28)**	**S 77.1% (37)** R 22.9% (11)	**S 87.9% (211)** I 11.6% (28) R 0.4% (1)	**S 98.3% (59)** I 1.7% (1)	**S 84.5% (93)** I 14.5% (16) R 1.0% (1)
*E coli gyrA (S83A)^b^*	Fluroquinolone	3.6% (24)	**48.0% (71)**	4.7% (11)							
*E coli gyrA (S83L)^b^*	Fluroquinolone	**33.4% (220)**	3.3% (5)	**36.3% (85)**							
*E coli gyrA (DA7G)^b^*	Fluroquinolone	**14.4% (95)**	0.0% (0)	**18.4% (43)**	Ciprofloxacin	**S 100.0% (134)**	**S 100.0% (44)**	**S 100.0% (48)**	**S 94.2% (226)** I 5.8% (14)	**S 100.0% (60)**	**S 90.0% (99)** I 10.0% (11)
*E coli gyrA (D87N)^b^*	Fluroquinolone	**16.3% (107)**	0.0% (0)	**35.0% (82)**							
*E coli gyrA (D87Y)^b^*	Fluroquinolone	2.1% (14)	3.3% (5)	0.0% (0)							

^*a*^ S = Susceptible, R = Resistant, I = Intermediate; based on 2018 CLSI guidelines.

^*b*^ Intrinsic AMR gene.

^*c*^ GEMS AMR data was from Table S2, Bengtsson *et al.* 2022 (6).

^*d*^ GEMS and VIDA disk diffusion results were provided by Tennant Lab, VIDA; Dataset 4 (9).

^*e*^ The bold formatting indicates differences in prevalence among the genes identified in the table.

^*f*^ N/A, not examined.

As our study contains *Sf*6 genomes not limited to GEMS, we examined the genomic distribution of ARGs among *Sf*6 alone (Data set 4). The *bla*_OXA-1_ gene encodes a class D β-lactamase that confers resistance to penicillin (42). Within GEMS, the presence of this gene was more common in non-*Sf*6 genomes (64.3%) than *Sf*6 (18.7%) (Table 4). Across geographically diverse *Sf*6 genomes, *bla*_OXA-1_ was present in 34% (109/320) of all *Sf*6 but was disproportionately present in *Sf*6 strains from Asia. The *bla*_OXA-1_ gene was identified in 53% (102/194) of *Sf*6 from Asia, but only 6% (7/126) of *Sf*6 from Africa. Other ARGs were found more frequently in *Sf*6 strains from countries in Asia included *catA1, sat2*, *tetB*-like*,* and *dfrA1* (Table 4).

The VIDA study compared the susceptibility of *S. flexneri* strains from both GEMS and VIDA sites in Africa (9). We performed a retrospective analysis of the serotype distribution patterns between non-*Sf*6, *Sf*2a, and *Sf*6 strains from Africa in GEMS and VIDA studies, anticipating that the genomic differences in ARG would cause phenotypic susceptibility differences (Data set 4).

Consistent with species levels analyses (9), non-*Sf*6, *Sf*2a, and *Sf*6 strains from Africa were susceptible to azithromycin and ciprofloxacin while resistant to trimethoprim–sulfonamide in both studies (Table 4). As with *bla*_OXA-1_ genomic distribution among GEMS strains (Table 4), we observed that 84% (112/134) of non-*Sf*6 strains from GEMS were resistant to ampicillin, while all *Sf*6 were susceptible. This resistance pattern was similarly distributed in non-*Sf*6 (73%; 176/240) and *Sf*6 (8%; 50/60) from VIDA. Of the non-*Sf*6, >95% of *Sf*2a strains from GEMS and VIDA were resistant to ampicillin (Table 4). These data supported the previous serotype-specific genomic distribution observed for *bla*_OXA-1_ and highlights serotype-specific resistance patterns within Africa.

### Geographically diverse *Sf*6 demonstrates phenotypic conservation that varies from *S. flexneri* archetype strain 2457T

**Fig 3:**
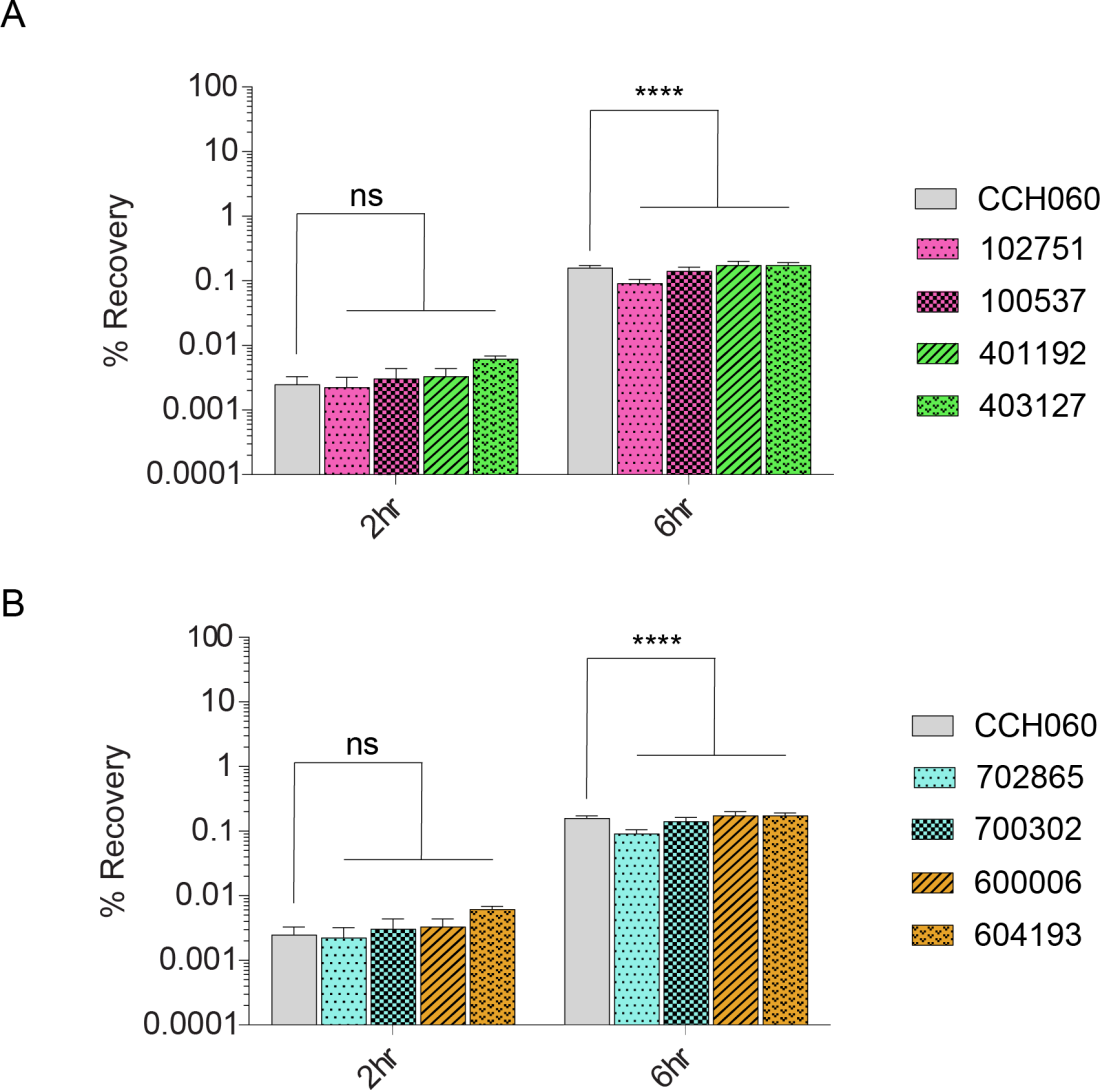
Invasion of geographically diverse *Sf*6 strains in HT-29 cells. GEMS *Sf6* clinical isolates from Africa (A) and Asia (B) and control archetype strain CCH060 were used to infect HT-29 monolayers (MOI of 1:100) for 90 min. Cells were lysed to enumerate intracellular bacteria at 2 and 6 hpi. Results are displayed as percent recovery of initial inoculum and shown for a representative assay of three replicates. The asterisks above indicate statistically significant differences determined using a two-way ANOVA and Tukey posttest; ****, *P* < 0.0001.

We previously determined that archetype *Sf*6 strain CCH060 invaded HT-29 cells at a lower rate than *Sf*2a and *Sf*3a archetype strains (18). Here, we assessed invasion and intracellular replication of geographically diverse *Sf*6 isolates from four different countries (The Gambia, Kenya, Pakistan, and Bangladesh) compared with archetype control strains *Sf*6 strain CCH060 and *Sf*2a strain 2457T using the gentamicin protection assay. At 2- and 6-h post-infection (hpi), strains from Africa (Fig. 3A) and Asia (Fig. 3B) had a similar number of intracellular bacteria recovered compared with *Sf*6 archetype strain CCH060. While similar to one another, all of the *Sf*6 strains compared had a reduced intracellular recovery as compared with *S. flexneri* strain 2457T (Fig. S1), as previously reported with archetype strain comparisons alone (18). The intracellular recovery of the *Sf*6 strains at 2 and 6 hpi was ~10-fold less than *S. flexneri* strain 2457T.

**Fig 4:**
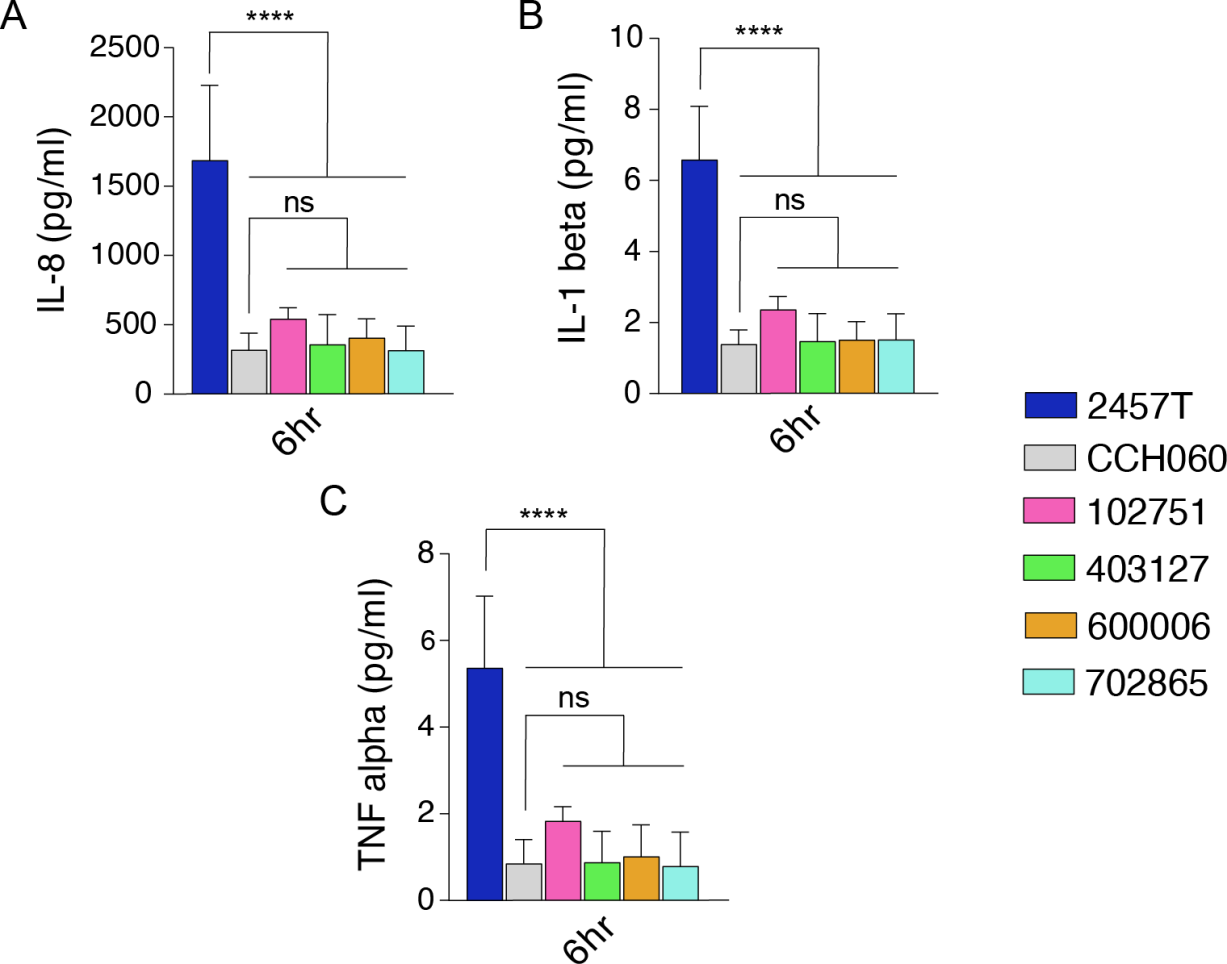
Multiplex cytokine comparison of *Sf*6 and *Sf2a* strain 2457T at 6 hpi HT-29 infection. Supernatants from infected HT-29 cells were collected at 2 and 6 hpi and assessed by multiplex-cytokine array. Cytokines tested included interleukins IL-8 (A), IL-1β (B), and tumor necrosis factor-alpha (TNF-α; C). Data are normalized by subtraction of uninfected control wells. Data are presented as pooled data from two independent experiments with technical triplicates in each experiment. The asterisks above indicate statistically significant differences between *Sf*6 isolates as compared to *Sf2a* 2457T or *Sf*6 CCH060 and were determined using a two-way ANOVA and Tukey posttest; ****, *P* < 0.0001.

Using a multi-array cytokine panel, we determined the levels of interferon-gamma (IFN-γ), tumor necrosis factor-alpha (TNF-α), and interleukins IL-1β, IL-2, IL-4, IL-6, IL-8, IL-10, IL-12p70, and IL-13 in tissue culture supernatants of HT-29 cells at 2 and 6 hpi infected with *Sf*6 strains (Fig. 4); *Sf*2a strain 2457T was used for comparison. At 2 hpi, all cytokines assessed were undetectable. At 6 hpi, IFN-γ, IL-2, IL-4, IL-10, IL-8, IL-12p70, and IL-13 were undetectable. IL-6, an essential pro-inflammatory cytokine for immune-epithelial crosstalk (43), while induced at low levels, showed no significant variation between the strains examined (data not shown). A similar amount of IL-8, a common cytokine associated with *Shigella* infection (44), was secreted by HT-29 cells infected with *Sf*6 strains (387 ± 166 pg/mL) but was 4.4-fold less than *S. flexneri* 2457T (1,687 ± 541 pg/mL) (Fig. 4A). Supernatants from *Sf*6-infected cells had ~fourfold less IL-1β and TNF-α compared with *S. flexneri* 2457T-infected cells (Fig. 4B and C). These results demonstrated a reduced capacity of *Sf*6 strains to induce inflammatory cytokines.

We previously demonstrated that secretion of IpaB and IpaC was reduced for *Sf*6 CCH060 compared with *Sf2a* 2457T, and IpaD levels were too low to compare by densitometry (18). Due to the high genomic similarity among *Sf*6 strains, we hypothesized that other *Sf*6 strains would exhibit a similarly reduced Ipa protein effector secretion. To examine Ipa protein distribution across representative *Sf*6 isolates, we performed a Western blot of whole cell lysate and supernatant fractions, comparing *Sf*6 strains to archetype control strains. All strains were treated with either deoxycholate (bile salt) or Congo Red (CR) to induce T3SS effector secretion (45). We demonstrated that bile salts do not impact the invasion capacity of *Sf*6 clinical isolates (Fig. S2), in agreement with previous findings (18).

**Fig 5:**
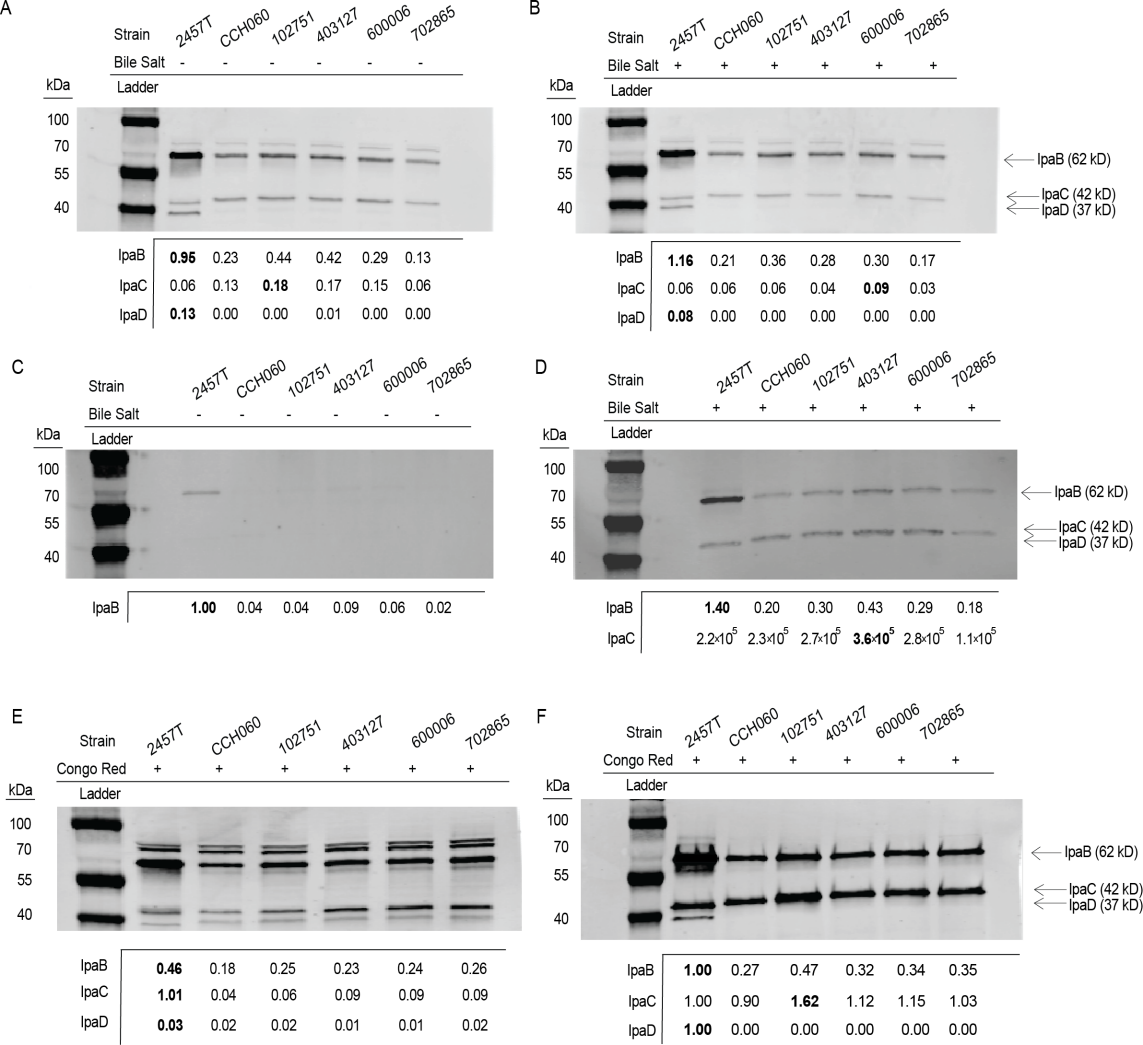
*Sf6* strains have reduced whole cell and secreted levels of Ipa protein expression compared with *Sf2a* 2457T. Whole cell lysates (A and B) and supernatant fractions (C and D) were collected from mid-log phase cultures grown in TSB (-) and TSB with deoxycholate (Bile Salt +) growth conditions and were probed for IpaB, IpaC, and IpaD with a mixture of specific antibodies by Western blot. GroEL was used as a loading control for whole cell lysates (Figure 3.S3 and 3.S4). Congo red (CR) induction was used as an alternative T3SS secretion stimulant for whole cell lysates (E) and supernatants (F). Densitometry (Fiji ImageJ) was utilized to compare protein levels (47).

Western blot and densitometry analysis of bile salt-treated bacteria demonstrated that all *Sf*6 strains expressed similar amounts of IpaB and IpaC in whole cell lysates and supernatant fractions (Fig. 5A through D; Fig. S3). In both whole cell lysate and secreted fractions, IpaB was reduced in *Sf*6 compared with *Sf*2a 2457T. IpaD was undetectable in supernatant fractions, but in whole cell lysates IpaD was detectable in *Sf*2a 2457T but not in any *Sf*6. Congo red induction resulted in similar IpaB and IpaC profiles as bile salt induction but allowed visualization of IpaD in supernatant (Fig. 5E and F; Fig. S4)(46). Secreted IpaD fractions were reduced for *Sf*6 compared with *Sf*2a 2457T (Fig. 5F). Reduced IpaB and IpaD expression was confirmed in concentrated supernatant fractions of *Sf*6 assessed by total protein staining (Fig. S5). This ensured that the observed reduction in Ipa protein secretion was not a result of reduced antibody binding efficiency to *Sf*6 T3SS effectors,

## DISCUSSION

*Sf6* continues to be one of the most prevalent *S. flexneri* serotypes recovered from children with MSD in LMICs (6, 10). This *S. flexneri* serotype has been under-characterized genomically (16, 17) and phenotypically (18, 48). Our previous study of archetype strains demonstrated that *Sf6* strain CCH060 contained unique genomic features and previously undescribed variation in key virulence phenotypes, such as invasion and T3SS effector secretion (18). In the current study, we expanded our analysis to include additional phenotypic assays, as well as a collection of geographically diverse *Sf*6 isolates. While genomically distant from the other *S. flexneri* serotypes, lying outside of the PG 1–7 schema, which do not include *Sf*6 (5), our data highlight a significant genomic similarity among all *Sf*6 strains examined, irrespective of isolation date or geography. This is not observed in other *S. flexneri* serotypes, where greater genome diversity is observed. This critical finding has implications for the development of preventative and therapeutic interventions, as well as diagnostics. The conservation among *Sf*6 strains supports the ability of a single *Sf*6 component in a multi-component *Shigella* vaccine to provide broad protection against circulating *Sf*6 isolates.

Genomic analyses between *Sf*6 strains demonstrated genomic similarity between strains obtained from the same geographic region. A recent study examining only *Sf*2a isolates from Sub-Saharan Africa identified limited geographic distribution of *Sf*2a strains across nine countries that share close, and in some cases adjacent borders (49). Comparatively, our study includes strains with wide geographic and temporal ranges of isolation. Irrespective of which study the *Sf*6 isolates were taken from or the timeframe in which they were obtained, *Sf*6 recovered from a particular country grouped together. Within each phylogenomically grouped geographic region, there remains genomic differences; however, that variation is significantly less than when *Sf*6 strains are compared with other *S. flexneri* serotypes.

The specific genetic features that distinguish *Sf*6 from the other *S. flexneri* had remained uncharacterized. Our analyses identified 1,211 genes conserved within all *Sf*6 genomes when compared with non-*Sf*6 strains. This included several metabolic genes, intrinsic ARGs, and genes encoding hypothetical proteins, as well as variation among several established *Shigella* virulence features. Of particular interest are the T2SS genes, which were present exclusively in all *Sf*6 strains. In *E. coli*, a close relative to *Shigella* (50), the T2SS is involved in translocation of many proteins to the extracellular space, including many virulence-related toxins and proteases (51). The T2SS is wide-spread among Gram-negative bacteria (51) but has not been previously identified in *S. flexneri,* as these genes are absent from the non-*Sf*6 genomes (27). There are three genes (*gspC, gspD*, and *gspE*) within the current *Sf*6 annotation for the T2SS gene cluster that appear to be pseudogenes; however, recent studies have demonstrated that pseudogene annotations in *S. flexneri* can be functional (52), and genomic assemblies often indicate inflated numbers of pseudogenes in *Shigella* (53). *Shigella* as a species have evolved through loss of genomic material (54, 55), so the retention of the more than 13 T2SS genes among *Sf6* suggests that the T2SS is a potential *Sf*6-specific virulence factor. A functional T2SS would potentially allow secretion of virulence-related peptides and proteases as in *E. coli* (51), or this secretion system may be functional in other phases of *Shigella* growth and replication, not associated with the human host. The function of a T2SS and the potential secreted proteins in any *Shigella* spp*.* are currently uncharacterized (27) and will require further functional investigation.

Another feature that distinguishes *Sf*6 from the other *S. flexneri* is the pINV. We discovered that the *Sf*6 pINV on average is ~38 kb smaller than the non-*Sf6* pINV, in line with early single-strain comparisons (22, 23). Comparative genomic analyses identified several putative virulence genes (*ospG*, *sepA*, *phon1*) and hypothetical genes from pWR100 that were absent from all *Sf*6 pINV plasmids. Within *Sf*6 genomes, the *ushA* gene encoded on pINV (33) was absent from *Sf*6 strains obtained in Bangladesh. The impact of the loss of putative virulence genes on *Sf*6 isolates, is unknown; however, the *Sf*6 as a serotype is well represented in epidemiological studies (3, 6, 8–10), suggesting that the loss of these genes and gene products does not significantly hinder human infection or dissemination in human populations. Together, these data highlight significant serotype-specific differences between pINV plasmids and support the need for further functional characterization, which may reveal novel information regarding plasmid–chromosome crosstalk and virulence kinetics in *Shigella* and specifically *Sf*6.

With the increasing prevalence of antibiotic resistance among *Shigella* (41), consistent surveillance and antibiograms are needed to inform local and global prescribing patterns. Our analysis identified differences in the distribution of ARGs by serotype and within geographic location of *Sf*6. Non-*Sf*6 GEMS strains contained more acquired ARGs than *Sf*6, including *bla*_OXA-1_. The *bla*_OXA-1_ gene, which encodes a beta-lactamase that can confer resistance to penicillin, a recommended antibiotic for shigellosis treatment (56), was present more often in *Sf*2a GEMS strains than *Sf*6. Phenotypic susceptibility testing from GEMS and VIDA strains in Africa revealed that non-*Sf*6 strains are often more resistant to ampicillin, while *Sf*6 are predominately susceptible. While disk diffusion was not performed on isolates from Asia, the genomic distribution of *bla*_OXA-1_ among *Sf*6 strains supports differential a geographic distribution of ampicillin resistance within this region as only three of the seven countries (Pakistan, Vietnam, and Thailand) contained *bla*_OXA-1_. This suggests ampicillin resistance may be spreading among *Sf*6 strains in Asia while still largely absent from *Sf*6 strains in Africa, which may be due to variation in prescribing patterns and antibiotic availability in these two continents.

The phenotypes previously identified for *Sf*6 strain CCH060, including reduced invasion, IL-8 production, and Ipa secretion were confirmed in a larger diverse set of *Sf*6 isolates. The loss of immune modulatory genes, such as *ospG*, the detection of conserved SNPs in established *Shigella* virulence genes (T3SS), and/or the presence of unique genomic features could be playing a role in these phenotypes. The previously identified sequence differences in *ipaB*, *ipaC*, and *ipaD* in archetype strain CCH060, as compared to *Sf*2a strain 2457T (18), were conserved in all 325 Sf6 genomes. Furthermore, we discovered that 97% (300/310) of *Sf*6 shared 100% nucleotide identity to one another for the entire *mxi-spa-ipa* loci (Table S2). Experiments are underway to explore how these genetic differences may affect virulence phenotypes for *Sf*6.

In conclusion, the results of our study demonstrate a remarkably high level of genomic and phenotypic conservation between geographically diverse *Sf*6 strains, which has not been documented for other *S. flexneri* serotypes. These data serve as important information for the development of diagnostic tools and preventative and therapeutic interventions.

## MATERIALS AND METHODS

### Bacterial strains and growth conditions

*S. flexneri* isolates were grown in tryptic soy broth (TSB; logarithmic) or on tryptic soy media (TSA) containing agar (BD Difco). Congo red (Sigma) was added to TSA media at a final concentration of 0.01% (w/v) and supplemented with 0.005% guanine to make CR-TSA plates (48). Bile salt-induced strains were grown in tryptic soy broth (Sigma) containing 0.1% (wt/vol) deoxycholate (DOC; [Sigma-Aldrich]) (57).

### Phylogenomic analysis

The genomes of 325 *S. flexneri* serotype 6 collected from three studies; 146 strains from GEMS (3, 6, 8, 58), 96 strains from other Asian countries (17), 56 strains from VIDA (9), 24 strains from Enterobase (19, 20), 2 from GenBank, and our CCH060 reference isolate (18). A total of seven strains were omitted due to distant phylogenomic grouping and potential mistyping as determined by phylogenomic analysis: six GEMS strains and one VIDA (Data set 1). Enterobase strains (https://enterobase.warwick.ac.uk/) were selected based on references used in previous phylogenomic analyses from public health labs. Clinical and routine strains from Enterobase with no metadata to determine travel and geographic origin of the *Sf*6 infection were omitted. SNPs were determined against the reference genome using NASP v1.2.0 (59) and the maximum-likelihood phylogeny using IQ-TREE 2 (60), with parameters as previously described (18). The phylogeny was midpoint-rooted and decorated using iTOL (61) and Adobe Illustrator. SNP distance was determined using snp-dists (https://github.com/tseemann/snp-dists; Data set 1).

#### *Sf6* vs *E. coli* and *Shigella* references

The *Sf*6 strains were compared with 73 previously sequenced *S. flexneri* genomes and 37 diverse *E. coli* and *Shigella* spp*.* genomes (26) (Data set 1); 158,917 SNPs were detected using snp-dists (https://github.com/tseemann/snp-dists). SNPs were determined against reference genome *E. coli* strain IAI39 (NC_011750.1)

#### *Sf6* vs *Sf6* genomes

SNPs were determined against reference genome CCH060 (CP099865, CP099866, CP09867); 6,904 SNPs were detected using snp-dists (https://github.com/tseemann/snp-dists). Phylogenetic analysis with Bayesian Analysis of Population Structure (BAPS v6.0) was performed to identify the clades of the *Sf*6 analysis in Fig. 1B (25).

### Genome sequencing and assembly

The completed *S. flexneri* serotype 6 strains (Table 1) were grown in lysogeny broth (LB) overnight, and their DNA was purified using a modified alkaline lysis and phenol–chloroform extraction method (62). The purified DNA was used to construct libraries and was sequenced on the Illumina HiSeq 6000 and the Oxford Nanopore sequencing and were assembled as previously described (62). The 57 *Sf*6 VIDA strains were sequenced and assembled as previously described (6).

### Genome based comparisons

#### Roary/Scoary

All genomes used in subsequent analyses (Data set 1) were annotated via Prodigal (63) to generate *de novo* CDS then PROKKA v1.14.6 (64). The resulting General Feature Formats (GFFs) were analyzed to identify core and accessory genes using Roary v3.13.0 (65) and Scoary version 1.6.16 (66) to categorize pangenome associations with Bonferroni correction for multiple observations (Data set 2; Tables 2 and 3).

#### LS-BSR pINV

We investigated differences in the total gene content of the plasmid among the genomes of 11 completed *S. flexneri* serotype 6 clinical strains and 314 *S. flexneri* serotype 6 draft genome sequences using BLASTN large-scale BLAST score ratio (LS-BSR) analysis as previously described (39, 40) (Data set 1, Data set 3). Plasmids for each of the completed genomes were annotated using PROKKA, described above and utilized as gene call reference for LS-BSR analyses. The pINV cluster from pWR100 was aligned and compared with p401192_196 using Mauve v.2025.2.5 with standard alignment parameters (38).

#### LS-BSR virulence genes

Established *Shigella* virulence genes (18), pWR100 (AL391753), *Sf*6 p401192_196 T3SS genes, and *S. boydii* serotype 4 T2SS genes (CP000036.1) were utilized as gene call reference to compare virulence genes across 325 *Sf*6 strains and 728 non-*Sf*6 strains (Data set 3); presence and absence cutoffs were used as previously described (18) (Data set 3).

#### Antimicrobial resistance analysis of *Sf*6

Antibiotic resistance genes were detected in each of the *Sf6* genomes using the Resistance Gene Identifier (RGI) v.6.0.3 and Comprehensive Antibiotic Resistance Database (CARD) v. 3.2.8 (67) with the following parameters: -a DIAMOND -t contig -d wgs -g PRODIGAL. All perfect and strict hits were further examined (Data set 4).

### HT-29 cell cultivation and gentamicin protection assay

Human HT-29 (ATCC HTB-38) monolayers were cultured in DMEM (Corning) supplemented with 10% Fetalplex (Gemini) and 2% HEPES (Quality Biological) in 150 cm^2^ flasks (Corning). The cells were incubated in 5% CO_2_ at 37°C gentamicin protection assays were performed as previously described (18). Control archetype strains, previously characterized by our laboratory (18), were utilized as *S. flexneri* controls; *S. flexneri* serotype 6 strain CCH060 and *S. flexneri* serotype 2a strain 2457T. Biological replicates (Fig. S1, n=two and Fig. 3, *n* = 3) were performed comparing the clinical strains to archetype strains.

### Cytokines/chemokines

Using archetype strains 2457T and CCH060 as controls, supernatants were collected from HT-29 invasion assays at 2 and 6 hpi (Fig. S1). Triplicate technical replicates of biological duplicate samples (*n* = 2) were assessed for each strain and time point. Cytokines and chemokines were quantified using commercial electrochemiluminescence microarray (Meso Scale Diagnostic, Rockville, MD, United States) following the manufacturer’s instructions. Cytokine tested included interferon-gamma (IFN-γ), tumor necrosis factor-alpha (TNF-α), and interleukins IL-1β, IL-2, IL-4, IL-6, IL-8, IL-10, IL-12p70, and IL-13 from MSD kit U-PLEX Proinflam Combo 1, human. Uninoculated controls were subtracted from reported results to account for background cytokine signal. All cytokine levels were reported as the amount picogram secreted per milliliter (pg/mL).

### Western blot

#### Bile salt induction

Bile salt induction was performed as previously described (18). Four replicates of bile salt inductions were performed for Western blot analysis and a representative figure was selected for presentation (Fig. 5).

#### Congo Red induction

Red colonies were picked from overnight plates and resuspended in TSB to a concentration of ~6 × 10^7^ CFU/mL and incubated at 37°C for ~2 h to mid-log phase (OD_600_ of ~0.7; 2 × 10^8^ CFU/mL). Samples were normalized and resuspended in PBS or PBS with CR then incubated for 3 h at 37°C. Bacteria were centrifuged at 4,500*×g* for 10 min, and supernatants were filtered (0.2 µm pore size) to remove bacteria. Supernatant, whole cell lysates, total protein visualization, and Ipa protein visualization were performed as previously described (18). Two replicates of Congo Red inductions were performed for Western blot and a representative figure was selected (Fig. 5).

### Statistics

Invasion data and cytokine analysis data were displayed in GraphPad Prism version 10, and all graphical statistics were performed in GraphPad using two-way ANOVA with Tukey post-test. Standard deviation calculations were used for comparing collective serotype analyses throughout the analyses using Microsoft Excel. Values are represented as the mean ± standard deviation for genome size and SNP calculations.

## Data Availability

The complete *Sf*6 genomes have been deposited under BioProject number PRJNA1133736 in NCBI and *Sf*6 VIDA strains under BioProject number PRJEB71076 in ENA; strain-specific accession numbers are listed in Data set 1.

## SUPPLEMENTAL MATERIAL

Data set 1 mbio.02210-24-s0001.xlsx Phylogenomic SNP distances and strain lists.

Data set 2 mbio.02210-24-s0002.xlsx Scoary-only analyses.

Data set 3 mbio.02210-24-s0003.xlsx LS-BSR analyses.

Data set 4 mbio.02210-24-s0004.xlsx RGI/CARD *Sf*6 only, ARG analysis for GEMS, and disk diffusion results.

Figure S1 mbio.02210-24-s0005.tif MSD invasion results.

Figure S2 mbio.02210-24-s0006.tif Bile salt invasion results.

Figure S3 mbio.02210-24-s0007.tif Western blot controls.

Figure S4 mbio.02210-24-s0008.tif Western blot CR controls.

Figure S5 mbio.02210-24-s0009.tif Ten-milliliter CR induction concentration.

Supplemental material mbio.02210-24-s0010.docx Supplemental tables and figure legends.

ASM does not own the copyrights to Supplemental Material that may be linked to, or accessed through, an article. The authors have granted ASM a non-exclusive, world-wide license to publish the Supplemental Material files. Please contact the corresponding author directly for reuse.

## References

[1] GBD 2017 Causes of Death Collaborators. 2018. Global, regional, and national age-sex-specific mortality for 282 causes of death in 195 countries and territories, 1980-2017: a systematic analysis for the Global Burden of Disease Study 2017. Lancet 392:1736–1788. doi:10.1016/S0140-6736(18)32203-7 30496103 PMC6227606

[2] Liu J, Platts-Mills JA, Juma J, Kabir F, Nkeze J, Okoi C, Operario DJ, Uddin J, Ahmed S, Alonso PL, et al.. 2016. Use of quantitative molecular diagnostic methods to identify causes of diarrhoea in children: a reanalysis of the GEMS case-control study. Lancet 388:1291–1301. doi:10.1016/S0140-6736(16)31529-X 27673470 PMC5471845

[3] Livio S, Strockbine NA, Panchalingam S, Tennant SM, Barry EM, Marohn ME, Antonio M, Hossain A, Mandomando I, Ochieng JB, et al.. 2014. Shigella isolates from the global enteric multicenter study inform vaccine development. Clin Infect Dis 59:933–941. doi:10.1093/cid/ciu468 24958238 PMC4166982

[4] GBD 2017 Diarrhoeal Disease Collaborators. 2020. Quantifying risks and interventions that have affected the burden of diarrhoea among children younger than 5 years: an analysis of the Global Burden of Disease Study 2017. Lancet Infect Dis 20:37–59. doi:10.1016/S1473-3099(19)30401-3 31678029 PMC7340495

[5] Connor TR, Barker CR, Baker KS, Weill F-X, Talukder KA, Smith AM, Baker S, Gouali M, Pham Thanh D, Jahan Azmi I, Dias da Silveira W, Semmler T, Wieler LH, Jenkins C, Cravioto A, Faruque SM, Parkhill J, Wook Kim D, Keddy KH, Thomson NR. 2015. Species-wide whole genome sequencing reveals historical global spread and recent local persistence in Shigella flexneri. Elife 4:e07335. doi:10.7554/eLife.07335 26238191 PMC4522646

[6] Bengtsson RJ, Simpkin AJ, Pulford CV, Low R, Rasko DA, Rigden DJ, Hall N, Barry EM, Tennant SM, Baker KS. 2022. Pathogenomic analyses of Shigella isolates inform factors limiting shigellosis prevention and control across LMICs. Nat Microbiol 7:251–261. doi:10.1038/s41564-021-01054-z 35102306 PMC8813619

[7] Kotloff K.L, Riddle MS, Platts-Mills JA, Pavlinac P, Zaidi AKM. 2018. Shigellosis. Lancet 391:801–812. doi:10.1016/S0140-6736(17)33296-8 29254859

[8] Kotloff KL, Nataro JP, Blackwelder WC, Nasrin D, Farag TH, Panchalingam S, Wu Y, Sow SO, Sur D, Breiman RF, et al.. 2013. Burden and aetiology of diarrhoeal disease in infants and young children in developing countries (the global enteric multicenter study, GEMS): a prospective, case-control study. Lancet 382:209–222. doi:10.1016/S0140-6736(13)60844-2 23680352

[9] Kasumba IN, Badji H, Powell H, Hossain MJ, Omore R, Sow SO, Verani JR, Platts-Mills JA, Widdowson M-A, Zaman SMA, et al.. 2023. Shigella in Africa: new insights from the vaccine impact on diarrhea in Africa (VIDA) study. Clin Infect Dis 76:S66–S76. doi:10.1093/cid/ciac969 37074444 PMC10116563

[10] Levine MM, Kotloff KL, Barry EM, Pasetti MF, Sztein MB. 2007. Clinical trials of Shigella vaccines: two steps forward and one step back on a long, hard road. Nat Rev Microbiol 5:540–553. doi:10.1038/nrmicro1662 17558427 PMC3771495

[11] Timakov VD, Petrovskaya VG, Bondarenko VM, Khomenko NA. 1972. Genetic data concerning Shigella flexneri serotypes 5 and 6. Int J Syst Bacteriol 22:149–154. doi:10.1099/00207713-22-3-149

[12] Schliessmann DJ, Cooley WT, Rabin R. 1957. The Manchester variety of Shigella flexneri 6 isolated in Kentucky. Pub Health Rep (1896) 72:720–722. 13453635 PMC2031328

[13] Bowman FB. 1908. A series of cases of tropical infantile dysentery with a hitherto undescribed bacillus as the causitative agent. Phil J Sci 3:31–38.

[14] Dmitriev BA, Knirel YA, Sheremet OK, Shashkov AA, Kochetkov NK, Hofman IL. 1979. Somatic antigens of Shigella. The structure of the specific polysaccharide of Shigella newcastle (Sh. flexneri type 6) lipopolysaccharide. Eur J Biochem 98:309–316. doi:10.1111/j.1432-1033.1979.tb13190.x 381001

[15] Gekker VD, Ravitch-Birger ED, Belaya JA. 1965. The position of Newcastle bacteria in the classification of the shigellae. Int Bull Bacteriol Nomencl Taxon 15:133–138. doi:10.1099/00207713-15-3-133

[16] Shahnaij M, Amin MB, Hoque MM, Mondol AS, Rana KJ, Azmi IJ, Talukder KA. 2023. Characterization of Shigella flexneri serotype 6 strains isolated from Bangladesh and identification of a new phylogenetic cluster. J Bacteriol 205:e0040622. doi:10.1128/jb.00406-22 36927058 PMC10127597

[17] Mai S-N, Bodhidatta L, Turner P, Wangchuk S, Ha Thanh T, Voong Vinh P, Pham DT, Rabaa MA, Thwaites GE, Thomson NR, Baker S, Chung The H. 2021. The evolutionary history of Shigella flexneri serotype 6 in Asia. Microb Genom 7:000736. doi:10.1099/mgen.0.000736 34904947 PMC8767353

[18] Gabor CE, Hazen TH, Delaine-Elias BC, Rasko DA, Barry EM. 2023. Genomic, transcriptomic, and phenotypic differences among archetype Shigella flexneri strains of serotypes 2a, 3a, and 6. mSphere 8:e0040823. doi:10.1128/msphere.00408-23 37830809 PMC10732043

[19] Zhou Z, Alikhan NF, Mohamed K, Fan Y, Achtman M, Agama Study Group. 2020. The EnteroBase user’s guide, with case studies on Salmonella transmissions, Yersinia pestis phylogeny, and Escherichia core genomic diversity. Genome Res 30:138–152. doi:10.1101/gr.251678.119 31809257 PMC6961584

[20] Yassine I, Lefèvre S, Hansen EE, Ruckly C, Carle I, Lejay-Collin M, Fabre L, Rafei R, Clermont D, de la Gandara MP, Dabboussi F, Thomson NR, Weill F-X. 2022. Population structure analysis and laboratory monitoring of Shigella by core-genome multilocus sequence typing. Nat Commun 13:551. doi:10.1038/s41467-022-28121-1 35087053 PMC8795385

[21] Bajunaid W, Haidar-Ahmad N, Kottarampatel AH, Ourida Manigat F, Silué N, Tchagang CF, Tomaro K, Campbell-Valois F-X. 2020. The T3SS of Shigella: expression, structure, function, and role in vacuole escape. Microorganisms 8:1933. doi:10.3390/microorganisms8121933 33291504 PMC7762205

[22] Lan R, Lumb B, Ryan D, Reeves PR. 2001. Molecular evolution of large virulence plasmid in Shigella clones and enteroinvasive Escherichia coli. Infect Immun 69:6303–6309. doi:10.1128/IAI.69.10.6303-6309.2001 11553574 PMC98765

[23] Lan R, Stevenson G, Reeves PR. 2003. Comparison of two major forms of the Shigella virulence plasmid pINV: positive selection is a major force driving the divergence. Infect Immun 71:6298–6306. doi:10.1128/IAI.71.11.6298-6306.2003 14573649 PMC219609

[24] Noriega FR, Liao FM, Maneval DR, Ren S, Formal SB, Levine MM. 1999. Strategy for cross-protection among Shigella flexneri serotypes. Infect Immun 67:782–788. doi:10.1128/IAI.67.2.782-788.1999 9916090 PMC96386

[25] Tang J, Hanage WP, Fraser C, Corander J. 2009. Identifying currents in the gene pool for bacterial populations using an integrative approach. PLoS Comput Biol 5:e1000455. doi:10.1371/journal.pcbi.1000455 19662158 PMC2713424

[26] Hazen TH, Daugherty SC, Shetty AC, Nataro JP, Rasko DA. 2017. Transcriptional variation of diverse enteropathogenic Escherichia coli isolates under virulence-inducing conditions. mSystems 2:e00024-17. doi:10.1128/mSystems.00024-17 28766584 PMC5527300

[27] Yang F, Yang J, Zhang X, Chen L, Jiang Y, Yan Y, Tang X, Wang J, Xiong Z, Dong J, et al.. 2005. Genome dynamics and diversity of Shigella species, the etiologic agents of bacillary dysentery. Nucleic Acids Res 33:6445–6458. doi:10.1093/nar/gki954 16275786 PMC1278947

[28] Kim DW, Lenzen G, Page AL, Legrain P, Sansonetti PJ, Parsot C. 2005. The Shigella flexneri effector OspG interferes with innate immune responses by targeting ubiquitin-conjugating enzymes. Proc Natl Acad Sci U S A 102:14046–14051. doi:10.1073/pnas.0504466102 16162672 PMC1236552

[29] Nguyen T, Kim T, Ta HM, Yeo WS, Choi J, Mizar P, Lee SS, Bae T, Chaurasia AK, Kim KK. 2019. Targeting mannitol metabolism as an alternative antimicrobial strategy based on the structure-function study of mannitol-1-phosphate dehydrogenase in Staphylococcus aureus. MBio 10:e02660-18. doi:10.1128/mBio.02660-18 31289190 PMC6623548

[30] Germani Y, Sansonetti PJ. 2006. The genus *Shigella*, p 99–122. In Dworkin M, Falkow S, Rosenberg E, Schleifer KH, Stackebrandt E (ed), The prokaryotes: a handbook on the biology of bacteria volume 6: proteobacteria: gamma subclass. Springer, New York.

[31] Andrews WH, Jacobson A. 2023. Shigella, bacteriological analytical manual, chapter 6, on FDA. Available from: https://www.fda.gov/food/laboratory-methods-food/bam-chapter-6-shigella. Retrieved 05 Mar 2024.

[32] Clayton FH, Warren SH. 1929. An unusual Bacillus recovered from cases presenting symptoms of dysentery. J Hyg (Lond) 28:355–362. doi:10.1017/s0022172400009670 20475005 PMC2167746

[33] Buchrieser C, Glaser P, Rusniok C, Nedjari H, D’Hauteville H, Kunst F, Sansonetti P, Parsot C. 2000. The virulence plasmid pWR100 and the repertoire of proteins secreted by the type III secretion apparatus of Shigella flexneri. Mol Microbiol 38:760–771. doi:10.1046/j.1365-2958.2000.02179.x 11115111

[34] Burns DM, Beacham IR. 1986. Nucleotide sequence and transcriptional analysis of the E. coli ushA gene, encoding periplasmic UDP-sugar hydrolase (5'-nucleotidase): regulation of the ushA gene, and the signal sequence of its encoded protein product. Nucleic Acids Res 14:4325–4342. doi:10.1093/nar/14.10.4325 3012467 PMC339864

[35] Maldonado-Contreras A, Birtley JR, Boll E, Zhao Y, Mumy KL, Toscano J, Ayehunie S, Reinecker H-C, Stern LJ, McCormick BA. 2017. Shigella depends on SepA to destabilize the intestinal epithelial integrity via cofilin activation. Gut Microbes 8:544–560. doi:10.1080/19490976.2017.1339006 28598765 PMC5730386

[36] Maurelli AT, Baudry B, d’Hauteville H, Hale TL, Sansonetti PJ. 1985. Cloning of plasmid DNA sequences involved in invasion of HeLa cells by Shigella flexneri. Infect Immun 49:164–171. doi:10.1128/iai.49.1.164-171.1985 2989179 PMC262074

[37] Niu C, Shang N, Liao X, Feng E, Liu X, Wang D, Wang J, Huang P, Hua Y, Zhu L, Wang H. 2013. Analysis of soluble protein complexes in Shigella flexneri reveals the influence of temperature on the amount of lipopolysaccharide. Mol Cell Proteomics 12:1250–1258. doi:10.1074/mcp.M112.025270 23378524 PMC3650336

[38] Darling ACE, Mau B, Blattner FR, Perna NT. 2004. Mauve: multiple alignment of conserved genomic sequence with rearrangements. Genome Res 14:1394–1403. doi:10.1101/gr.2289704 15231754 PMC442156

[39] Sahl JW, Caporaso JG, Rasko DA, Keim P. 2014. The large-scale blast score ratio (LS-BSR) pipeline: a method to rapidly compare genetic content between bacterial genomes. PeerJ 2:e332. doi:10.7717/peerj.332 24749011 PMC3976120

[40] Hazen TH, Donnenberg MS, Panchalingam S, Antonio M, Hossain A, Mandomando I, Ochieng JB, Ramamurthy T, Tamboura B, Qureshi S, Quadri F, Zaidi A, Kotloff KL, Levine MM, Barry EM, Kaper JB, Rasko DA, Nataro JP. 2016. Genomic diversity of EPEC associated with clinical presentations of differing severity. Nat Microbiol 1:15014. doi:10.1038/nmicrobiol.2015.14 27571975 PMC5067155

[41] Shrivastava S, Shrivastava P, Ramasamy J. 2018. World health organization releases global priority list of antibiotic-resistant bacteria to guide research, discovery, and development of new antibiotics. J Med Soc 32:76. doi:10.4103/jms.jms_25_17

[42] Evans BA, Amyes SGB. 2014. OXA β-lactamases. Clin Microbiol Rev 27:241–263. doi:10.1128/CMR.00117-13 24696435 PMC3993105

[43] Guo Y, Wang B, Wang T, Gao L, Yang Z, Wang F, Shang H, Hua R, Xu J. 2021. Biological characteristics of IL-6 and related intestinal diseases. Int J Biol Sci 17:204–219. doi:10.7150/ijbs.51362 33390844 PMC7757046

[44] Sansonetti PJ, Arondel J, Huerre M, Harada A, Matsushima K. 1999. Interleukin-8 controls bacterial transepithelial translocation at the cost of epithelial destruction in experimental shigellosis. Infect Immun 67:1471–1480. doi:10.1128/IAI.67.3.1471-1480.1999 10024597 PMC96483

[45] Pope LM, Reed KE, Payne SM. 1995. Increased protein secretion and adherence to HeLa cells by Shigella spp. following growth in the presence of bile salts. Infect Immun 63:3642–3648. doi:10.1128/iai.63.9.3642-3648.1995 7642302 PMC173505

[46] Bahrani FK, Sansonetti PJ, Parsot C. 1997. Secretion of Ipa proteins by Shigella flexneri: inducer molecules and kinetics of activation. Infect Immun 65:4005–4010. doi:10.1128/iai.65.10.4005-4010.1997 9316999 PMC175575

[47] Schindelin J, Arganda-Carreras I, Frise E, Kaynig V, Longair M, Pietzsch T, Preibisch S, Rueden C, Saalfeld S, Schmid B, Tinevez JY, White DJ, Hartenstein V, Eliceiri K, Tomancak P, Cardona A. 2012. Fiji: an open-source platform for biological-image analysis. Nat Methods 9:676–682. doi:10.1038/nmeth.2019 22743772 PMC3855844

[48] DeLaine BC, Wu T, Grassel CL, Shimanovich A, Pasetti MF, Levine MM, Barry EM. 2016. Characterization of a multicomponent live, attenuated Shigella flexneri vaccine. Pathog Dis 74:ftw034. doi:10.1093/femspd/ftw034 27106253 PMC5985478

[49] Stenhouse GE, Keddy KH, Bengtsson RJ, Hall N, Smith AM, Thomas J, Iturriza-Gómara M, Baker KS. 2023. The genomic epidemiology of shigellosis in South Africa. Nat Commun 14:7715. doi:10.1038/s41467-023-43345-5 38001075 PMC10673971

[50] Sahl JW, Morris CR, Emberger J, Fraser CM, Ochieng JB, Juma J, Fields B, Breiman RF, Gilmour M, Nataro JP, Rasko DA. 2015. Defining the phylogenomics of Shigella species: a pathway to diagnostics. J Clin Microbiol 53:951–960. doi:10.1128/JCM.03527-14 25588655 PMC4390639

[51] Patrick M, Gray MD, Sandkvist M, Johnson TL. 2010. Type II secretion in Escherichia coli. EcoSal Plus 4. doi:10.1128/ecosalplus.4.3.4 26443782

[52] Chanin RB, Nickerson KP, Llanos-Chea A, Kumar DA, de la Parra DKV, Auclair J JR, Ding J, Li K, Dogiparthi SK, Kusber BJD, Faherty CS. 2019. Shigella flexneri adherence factor expression in in vivo-like conditions. mSphere 4:e00751-19. doi:10.1101/514679 31722995 PMC6854044

[53] Cooley NP, Wright ES. 2024. Many purported pseudogenes in bacterial genomes are bona fide genes. BMC Genomics 25:365. doi:10.1186/s12864-024-10137-0 38622536 PMC11017572

[54] Maurelli AT, Fernández RE, Bloch CA, Rode CK, Fasano A. 1998. “Black holes” and bacterial pathogenicity: a large genomic deletion that enhances the virulence of Shigella spp. and enteroinvasive Escherichia coli. Proc Natl Acad Sci U S A 95:3943–3948. doi:10.1073/pnas.95.7.3943 9520472 PMC19942

[55] Maurelli AT. 2007. Black holes, antivirulence genes, and gene inactivation in the evolution of bacterial pathogens. FEMS Microbiol Lett 267:1–8. doi:10.1111/j.1574-6968.2006.00526.x 17233672

[56] Shane AL, Mody RK, Crump JA, Tarr PI, Steiner TS, Kotloff K, Langley JM, Wanke C, Warren CA, Cheng AC, Cantey J, Pickering LK. 2017. 2017 infectious diseases society of America clinical practice guidelines for the diagnosis and management of infectious diarrhea. Clin Infect Dis 65:e45–e80. doi:10.1093/cid/cix669 29053792 PMC5850553

[57] Olive AJ, Kenjale R, Espina M, Moore DS, Picking WL, Picking WD. 2007. Bile salts stimulate recruitment of IpaB to the Shigella flexneri surface, where it colocalizes with IpaD at the tip of the type III secretion needle. Infect Immun 75:2626–2629. doi:10.1128/IAI.01599-06 17296762 PMC1865747

[58] Kotloff KL BW, Nasrin D, Nataro JP, Farag TH, van Eijk A, Adegbola RA, Alonso PL, Breiman RF, Faruque AS, Saha D, Sow SO, AK Z, Sur D, Zaidi AK, Biswas K, Panchalingam S, Clemens CD, Cohen D, Glass RI, Mintz ED, Sommerfelt H, Levine MM. 2018. Study: GEMS1 Case Control., ClinEpiDB rel. 5, 2018-DEC-13

[59] Sahl JW, Lemmer D, Travis J, Schupp JM, Gillece JD, Aziz M, Driebe EM, Drees KP, Hicks ND, Williamson CHD, Hepp CM, Smith DE, Roe C, Engelthaler DM, Wagner DM, Keim P. 2016. NASP: an accurate, rapid method for the identification of SNPs in WGS datasets that supports flexible input and output formats. Microb Genom 2:e000074. doi:10.1099/mgen.0.000074 28348869 PMC5320593

[60] Minh BQ, Schmidt HA, Chernomor O, Schrempf D, Woodhams MD, von Haeseler A, Lanfear R. 2020. IQ-TREE 2: new models and efficient methods for phylogenetic inference in the genomic era. Mol Biol Evol 37:1530–1534. doi:10.1093/molbev/msaa015 32011700 PMC7182206

[61] Letunic I, Bork P. 2021. Interactive tree of life (iTOL) v5: an online tool for phylogenetic tree display and annotation. Nucleic Acids Res 49:W293–W296. doi:10.1093/nar/gkab301 33885785 PMC8265157

[62] Hazen TH, Michalski J, Nagaraj S, Okeke IN, Rasko DA. 2017. Characterization of a large antibiotic resistance plasmid found in enteropathogenic Escherichia coli strain B171 and its relatedness to plasmids of diverse E. coli and Shigella strains. Antimicrob Agents Chemother 61:e00995-17. doi:10.1128/AAC.00995-17 28674052 PMC5571317

[63] Hyatt D, Chen GL, Locascio PF, Land ML, Larimer FW, Hauser LJ. 2010. Prodigal: prokaryotic gene recognition and translation initiation site identification. BMC Bioinformatics 11:119. doi:10.1186/1471-2105-11-119 20211023 PMC2848648

[64] Seemann T. 2014. Prokka: rapid prokaryotic genome annotation. Bioinformatics 30:2068–2069. doi:10.1093/bioinformatics/btu153 24642063

[65] Fu L, Niu B, Zhu Z, Wu S, Li W. 2012. CD-HIT: accelerated for clustering the next-generation sequencing data. Bioinformatics 28:3150–3152. doi:10.1093/bioinformatics/bts565 23060610 PMC3516142

[66] Brynildsrud O, Bohlin J, Scheffer L, Eldholm V. 2016. Rapid scoring of genes in microbial pan-genome-wide association studies with Scoary. Genome Biol 17:238. doi:10.1186/s13059-016-1108-8 27887642 PMC5124306

[67] Alcock BP, Huynh W, Chalil R, Smith KW, Raphenya AR, Wlodarski MA, Edalatmand A, Petkau A, Syed SA, Tsang KK, et al.. 2023. CARD 2023: expanded curation, support for machine learning, and resistome prediction at the comprehensive antibiotic resistance database. Nucleic Acids Res 51:D690–D699. doi:10.1093/nar/gkac920 36263822 PMC9825576

