# Supplementary figures and images for: Characterization of Shigella flexneri serotype 6 strains from geographically diverse low- and middle-income countries
Source: mBio. 2024 Dec 10;16(1):e02210-24. doi: 10.1128/mbio.02210-24 (PMC11708030; doi:10.1128/mbio.02210-24)

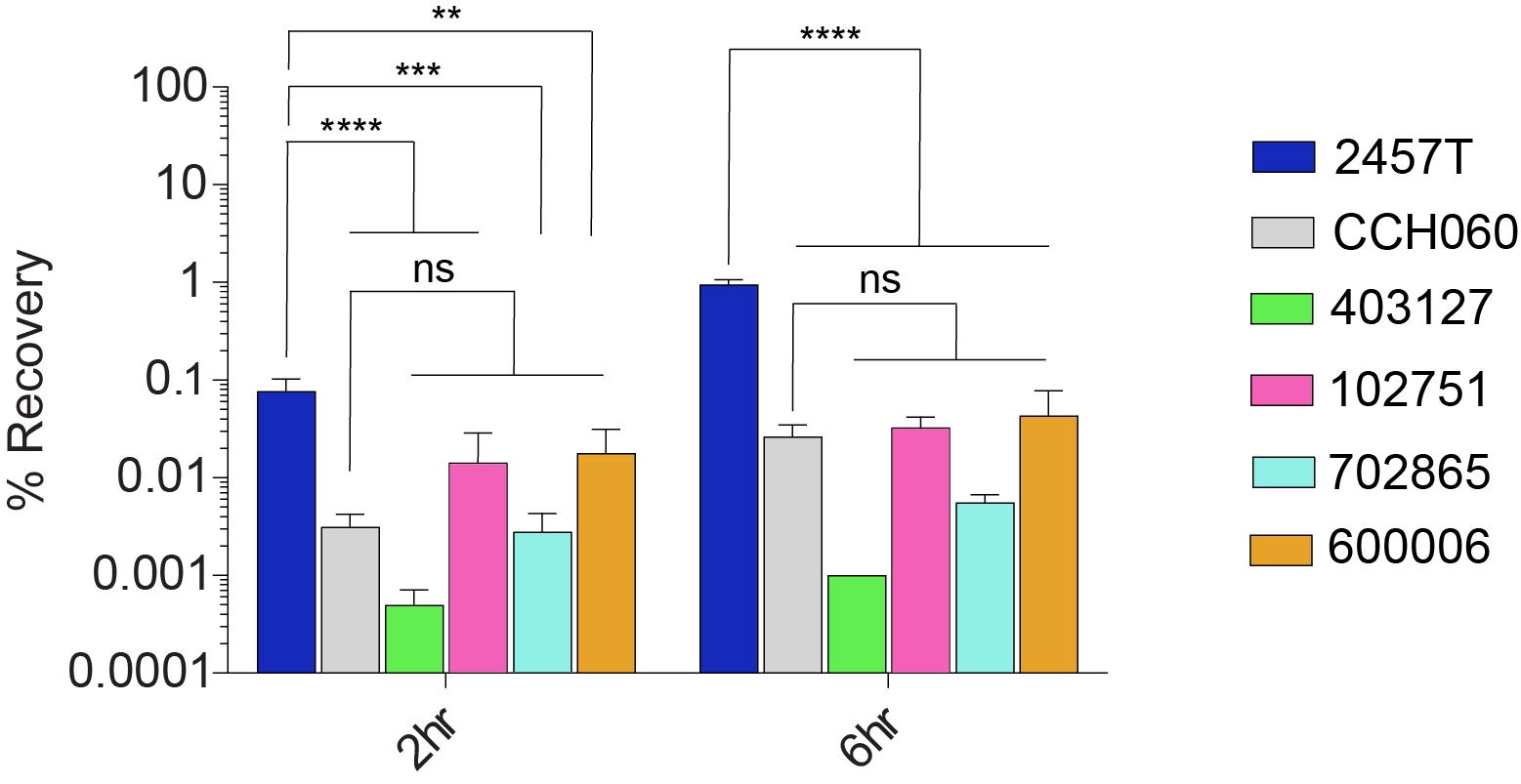

Supplement: Figure S1 — MSD invasion results. [file mbio.02210-24-s0005.tif]

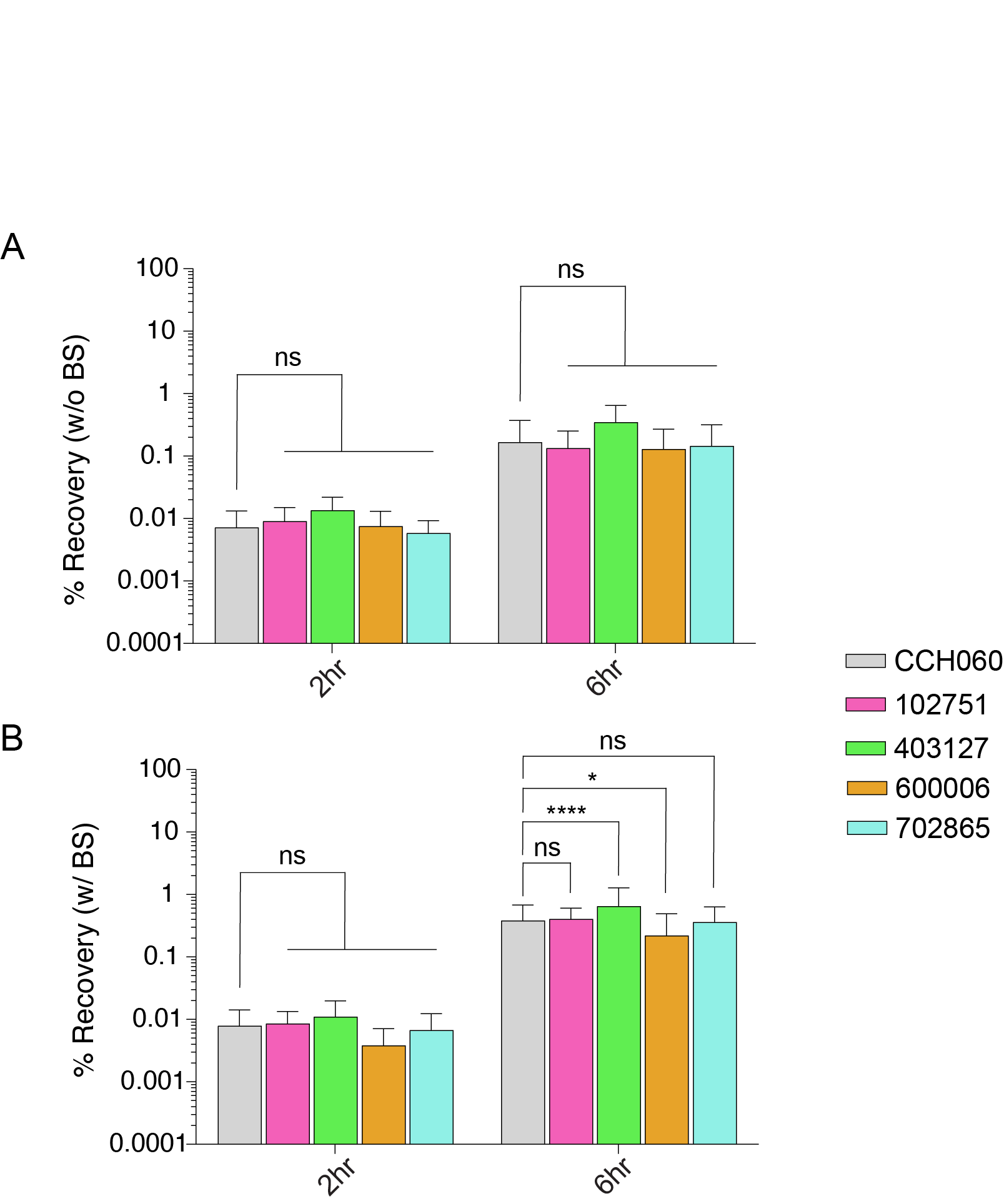

Supplement: Figure S2 — Bile salt invasion results. [file mbio.02210-24-s0006.tif]

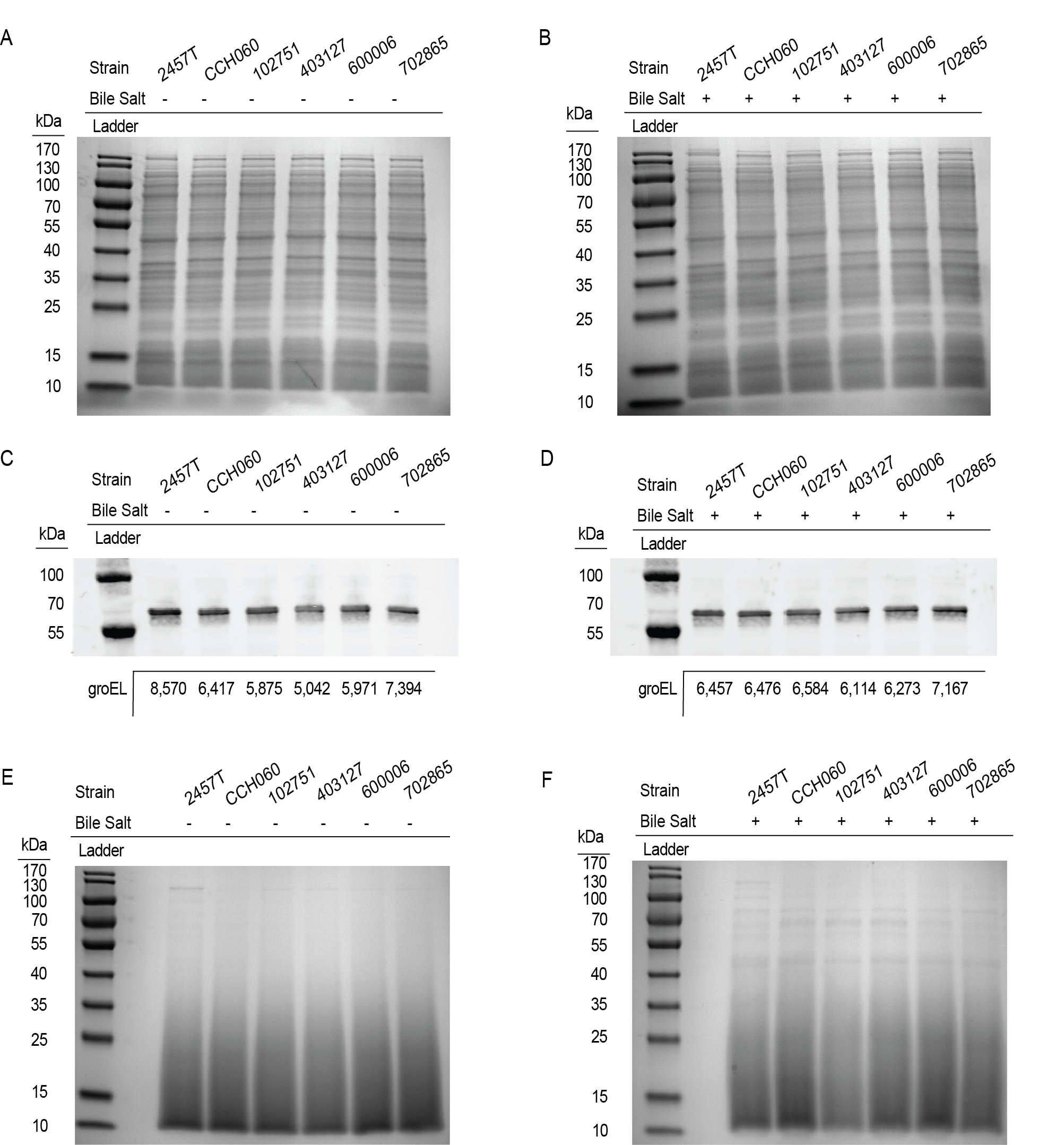

Supplement: Figure S3 — Western blot controls. [file mbio.02210-24-s0007.tif]

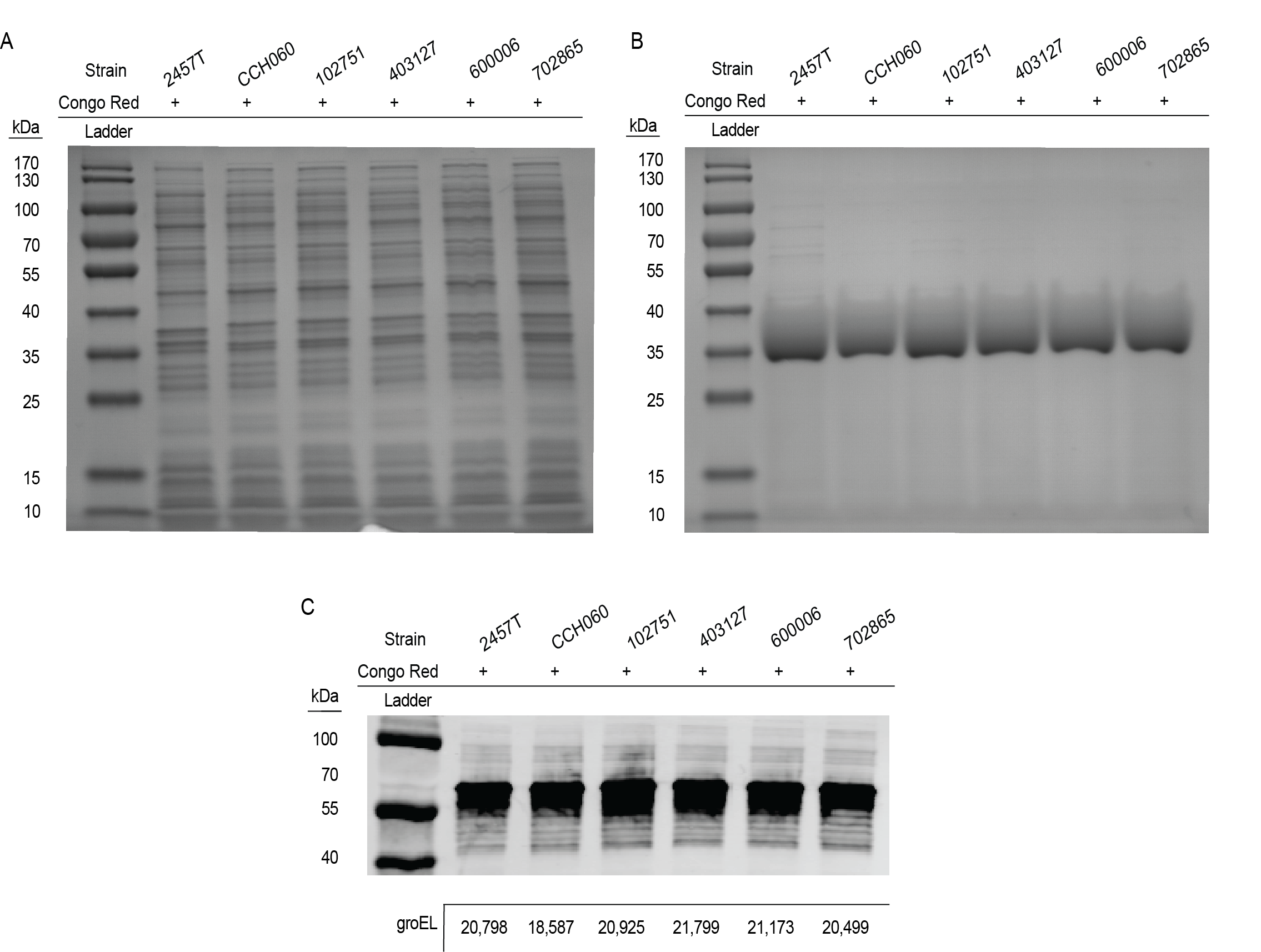

Supplement: Figure S4 — Western blot CR controls. [file mbio.02210-24-s0008.tif]

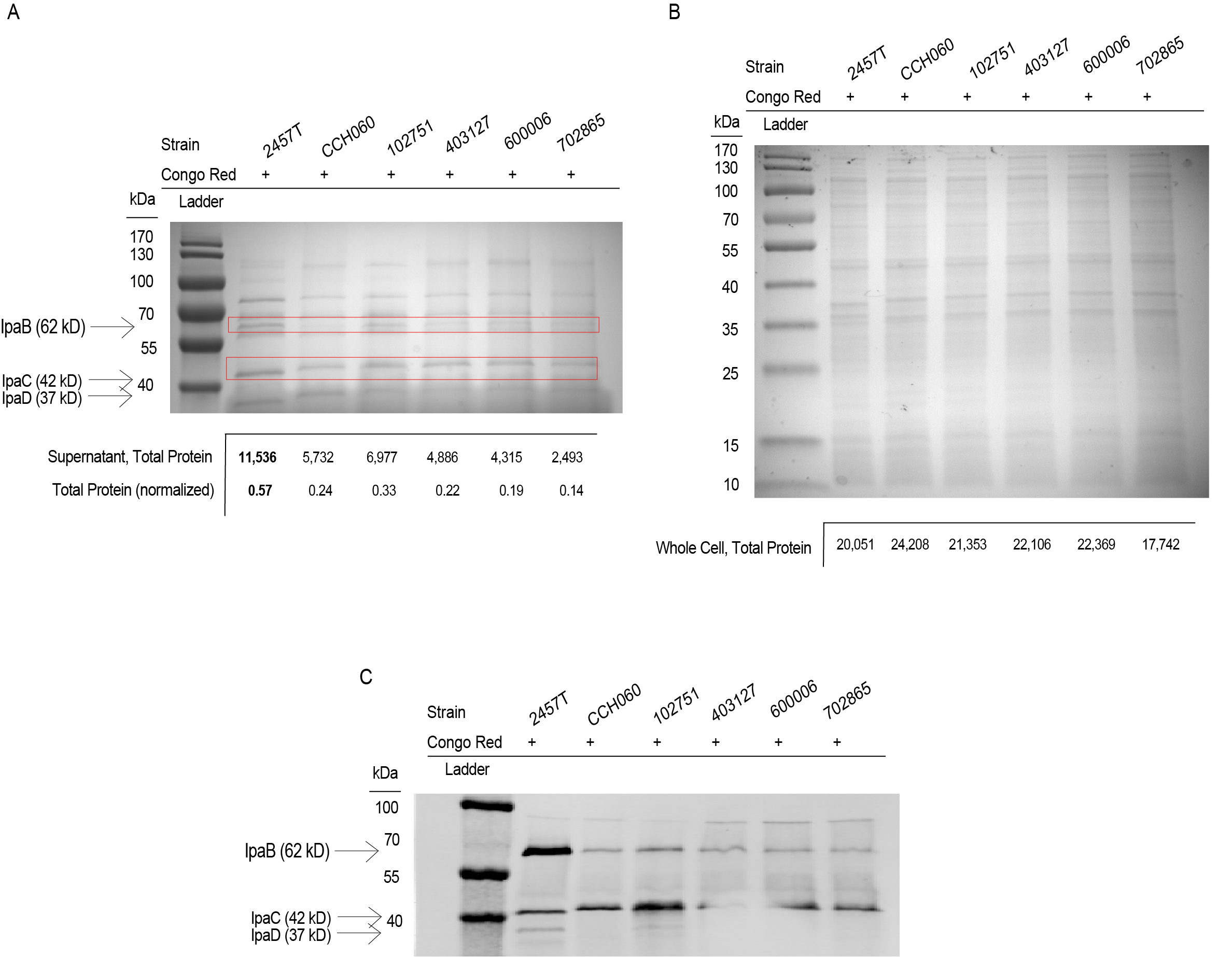

Supplement: Figure S5 — Ten-milliliter CR induction concentration. [file mbio.02210-24-s0009.tif]
