# Supplementary material for: Characterization of Shigella flexneri serotype 6 strains from geographically diverse low- and middle-income countries
Source: mBio. 2024 Dec 10;16(1):e02210-24. doi: 10.1128/mbio.02210-24 (PMC11708030; doi:10.1128/mbio.02210-24)
Supplement: Supplemental material — Supplemental tables and figure legends. [file mbio.02210-24-s0010.docx]

| **Table S1.** *S. flexneri* pINV and chromosome strain list | | | | | | |  | | |  | |  |
| --- | --- | --- | --- | --- | --- | --- | --- | --- | --- | --- | --- | --- |
| **Isolate** | | **Serotype** | | **pINV size (bp)** | | **Accession No.** | | | | **Chromosome size (bp)** | | **Accession No.** |
| 670 | | 1a | | 228,834 | | CP020087.1 | | | | 4,623,260 | | CP020086.1 |
| FDAARGOS_535 | | 1b | | 217,451 | | CP034059.1 | | | | 4,586,233 | | CP034060.1 |
| A-10383 | | 1b | | 218,629 | | CP130064.1 | | | | 4,576,609 | | CP130063.1 |
| SF-018-002 | | 1b | | 215,531 | | CP127082.1 | | | | 4,495,661 | | CP127081.1 |
| Y394 | | 1c | | 221,293 | | CP030773.1 | | | | 4,584,634 | | CP020753.1 |
| AUSMDU00010535 | | 2a | | 234,182 | | CP045942.1 | | | | 4,723,195 | | CP045941.1 |
| AUSMDU00008332 | | 2a | | 234,171 | | LR213456.1 | | | | 4,633,147 | | LR213455.1 |
| G1663 | | 2a | | 222,586 | | CP007038.1 | | | | 4,594,725 | | CP007037.1 |
| 2016AM-0877 | | 2a | | 222,142 | | CP033511.1 | | | | 4,719,160 | | CP033510.1 |
| 1508 | | 2a | | 222,126 | | CP030916.1 | | | | 4,696,723 | | CP030915.1 |
| AR-0425 | | 2a | | 222,114 | | CP044154.1 | | | | 4,538,615 | | CP044152.1 |
| 2457T | | 2a | | 221,974 | | CP100045.1 | | | | 4,643,553 | | CP100044.1 |
| 301 | | 2a | | 221,618 | | AF386526.1 | | | | 4,607,202 | | AE005674.2 |
| Sflex 21-42 | | 2a | | 225,592 | | CP121222.1 | | | | 4,644,259 | | CP121223.1 |
| FDAARGOS_716 | | 2b | | 231,172 | | CP050984.1 | | | | 4,751,732 | | CP050985.1 |
| FDAARGOS_690 | | 2b | | 228,903 | | CP055137.1 | | | | 4,674,361 | | CP055138.1 |
| J17B | | 3a | | 264,890 | | CP100043.1 | | | | 4,709,683 | | CP100042.1 |
| AUSMDU00021847 | | 3a | | 233,332 | | LR861786.1 | | | | 4,522,850 | | LR861785.1 |
| AUSMDU00022017 | | 3a | | 233,274 | | LR861789.1 | | | | 4,522,608 | | LR861788.1 |
| 83 | | 3a | | 231,165 | | LR979366.1 | | | | 4,522,047 | | LR878366.1 |
| 888048 | | 3a | | 231,092 | | NZ_MW396862.1 | | | | 4,519,004 | | CP066809.1 |
| FDAARGOS_74 | | 3a | | 229,413 | | CP026099.1 | | | | 4,576,448 | | CP026098.1 |
| FDAARGOS_713 | | 3a | | 220,335 | | CP054891.1 | | | | 4,593,694 | | CP054892.1 |
| 2013C-3749 | | 3b | | 223,405 | | CP034933.1 | | | | 4,654,019 | | CP034931.1 |
| AUSMDU00008332 | | 4c | | 223,354 | | CP020337.1 | | | | 4,661,857 | | CP020336.1 |
| 1205 | | 4c | | 222,060 | | CP012142.1 | | | | 4,683,636 | | CP012140.1 |
| 74-1170 | | 5a | | 251,323 | | CP026794.1 | | | | 4,733,503 | | CP026793.1 |
| M90T | | 5a | | 232,195 | | CP037924.1 | | | | 4,596,714 | | CP037923.1 |
| 94-3007 | | 7b | | 220,282 | | CP024476.1 | | | | 4,533,699 | | CP024473.1 |
| 2002017 | | Fxv | | 223,364 | | CP001384.1 | | | | 4,650,856 | | CP001383.1 |
| M2091 | | unknown | | 224,303 | | CP058591.1 | | | | 4,682,307 | | CP058589.1 |
| 89-141 | | Y | | 245,004 | | CP026804.1 | | | | 4,481,548 | | CP026803.1 |
| AR-0424 | | Y | | 226,601 | | CP044157.1 | | | | 4,593,360 | | CP044155.1 |
| 93-3063 | | Y | | 220,759 | | CP026771.1 | | | | 4,628,330 | | CP026768.1 |
| ^a^ Publicly available *Sf*6 pINV genomes omitted (FDAARGOS_714, CCH060, and 64-5500) | | | | | | | | | |  | |  |
| ^b^ Publicly available non-*Sf*6 genomes omitted from pINV sizing due to missing *mxi-spa-ipa, virB*, and *virF* genes (strains 61-4982,ATCC 29903, 71-783, NCTC 9278, 439, 981, AR-0423, FDAARGOS_689, 71-2783, and pSF5) | | | | | | | | | |  | |  |
| **Table S2.** T3SS alignment of p401192_196 T3SS to archetype strains | | | | | | | | | |  |  |  |
|  | | 2457T | | | | | CCH060 | | |  |  |  |
|  | | Percent Identity | | SNPs | | | Percent Identity | SNPs | |  |  |  |
| *spa40* | | 99.42% | | 5 | | | 100% | 0 | |  |  |  |
| *spa29* | | 99.48% | | 4 | | | 100% | 0 | |  |  |  |
| *spa9* | | 98.85% | | 3 | | | 100% | 0 | |  |  |  |
| *spa24* | | 98.31% | | 11 | | | 100% | 0 | |  |  |  |
| *spa33* | | 99.43% | | 5 | | | 100% | 0 | |  |  |  |
| *spa32* | | 100% | | 0 | | | 100% | 0 | |  |  |  |
| *spa13* | | 97.91% | | 1 | | | 100% | 0 | |  |  |  |
| *spa47* | | 99.23% | | 10 | | | 99.92% | 1 | |  |  |  |
| *spa15* | | 99.50% | | 2 | | | 100% | 0 | |  |  |  |
| *mxiA* | | 99.85% | | 3 | | | 100% | 0 | |  |  |  |
| *mxiC* | | 99.53% | | 4 | | | 100% | 0 | |  |  |  |
| *mxiD* | | 99.41% | | 10 | | | 99.94% | 1 | |  |  |  |
| *mxiE* | | 99.84% | | 1 | | | 100% | 0 | |  |  |  |
| *mxiM* | | 99.77% | | 1 | | | 99.53% | 2 | |  |  |  |
| *mxiL* | | 99.51% | | 2 | | | 100% | 0 | |  |  |  |
| *mxiK* | | 99.57% | | 3 | | | 100% | 0 | |  |  |  |
| *mxiJ* | | 99.59% | | 3 | | | 100% | 0 | |  |  |  |
| *mxiI* | | 99.32% | | 2 | | | 100% | 0 | |  |  |  |
| *mxiH* | | 94.44% | | 14 | | | 100% | 0 | |  |  |  |
| *mxiG* | | 97.58% | | 27 | | | 100% | 0 | |  |  |  |
| *ipgF* | | 98.04% | | 9 | | | 100% | 0 | |  |  |  |
| *ipgE* | | 97.52% | | 9 | | | 100% | 0 | |  |  |  |
| *ipgD* | | 96.78% | | 52 | | | 100% | 0 | |  |  |  |
| *icsB* | | 98.18% | | 27 | | | 100% | 0 | |  |  |  |
| *ipgA* | | 99.74% | | 1 | | | 100% | 0 | |  |  |  |
| *ipgB1* | | 99.52% | | 3 | | | 100% | 0 | |  |  |  |
| *ipgC* | | 98.72% | | 6 | | | 100% | 0 | |  |  |  |
| *ipaB* | | 98.51% | | 26 | | | 100% | 0 | |  |  |  |
| *ipaC* | | 97.62% | | 26 | | | 100% | 0 | |  |  |  |
| *ipaD* | | 97.30% | | 27 | | | 100% | 0 | |  |  |  |
| *ipaA* | | 99.58% | | 8 | | | 100% | 0 | |  |  |  |
| *virB* | | 99.78% | | 2 | | | 100% | 0 | |  |  |  |

**Supplemental Figure Legends**

**Figure S1. Invasion assay of geographically diverse *Sf*6 strains from HT-29 cells as compared to archetype strain 2457T.** Geographically diverse *S. flexneri* serotype 6 GEMS clinical strains were compared to archetype strain 2457T. Strains were used to infect HT-29 monolayers (MOI of 1:100) for 90min. Cells were lysed to enumerate intracellular bacteria at 2h and 6h pi. Results are normalized by inoculum and displayed as percent recovery for a representative assay of two replicates. The asterisks above indicate statistically significant differences determined using a two-way ANOVA and Tukey posttest; **, *p* <0.01; ***, *p* <0.001; ****, *p* <0.0001

**Figure S2. Invasion of geographically diverse *Sf*6 strains with TSB and TSB containing deoxycholate.** GEMS *S. flexneri* serotype 6 clinical strains from Africa and Asia along with archetype control strain CCH060 infected HT-29 monolayers (MOI of 1:100) for 90min. Bacteria were grown to logarithmic phase in **(Panel** **A)** TSB or **(Panel B)** TSB containing 0.1% deoxycholate (BS) for 2hr prior to infection. Cells were lysed to enumerate intracellular bacteria at 2h and 6h pi. Results are normalized by inoculum and displayed as percent recovery for a representative assay of three replicates The asterisks above indicate statistically significant differences determined using a two-way ANOVA and Tukey posttest; *, *p* <0.05; ****, *p* <0.0001

**Figure S3.** **Protein analysis of supernatant and whole cell lysate in deoxycholate (bile salts) containing growth conditions.** Whole cell lysates and corresponding supernatant and were collected during TSB and TSB with deoxycholate growth conditions as outlined in materials and methods. Total protein was analyzed for whole cell lysates (**Panels A-B**) and supernatant (**Panels E-F**) using GelCode. Total protein analyses were performed to confirm equal loading of samples (Figure 5) and the effect of bile salt was in accordance with previous literature (44). **Panels C-D:** Western Blot to identify GroEL was used as a loading control to normalize whole cell lysates as quantified by densitometry (Fiji ImageJ).

**Figure S4.** **Protein analysis of whole cell lysate and** **supernatant in Congo Red (CR) containing growth conditions.** Whole cell lysates and supernatant were collected from PBS and PBS with CR conditions as described in material and methods. Total protein was analyzed for whole cell lysates (**Panel A**) and supernatant (**Panel B**) using GelCode. Total protein analyses was performed to confirm equal loading of samples (Figure 5) and CR effect was in accordance with previous literature (67). **Panel C:** Western Blot to identify GroEL was used as a loading control to normalize whole cell lysates as quantified by densitometry (Fiji ImageJ).

**Figure S5. Protein analysis of whole cell lysates and 10mL supernatant concentration in CR containing growth conditions.** Supernatant (**Panel A**) and Whole cell lysates (**Panel B**) were collected from PBS with CR conditions; 10ml of supernatant was concentrated as compared to 1ml of whole cell lysate. Total protein was analyzed using GelCode and quantified using densitometry (Fiji ImageJ). **Panel C**, Western blot analysis of IpaB, IpaC, and IpaD was performed on supernatant fractions from **Panel A.**
